# Solar-Driven Sustainability: III–V Semiconductor for Green Energy Production Technologies

**DOI:** 10.1007/s40820-024-01412-6

**Published:** 2024-07-11

**Authors:** Bagavath Chandran, Jeong-Kyun Oh, Sang-Wook Lee, Dae-Young Um, Sung-Un Kim, Vignesh Veeramuthu, Jin-Seo Park, Shuo Han, Cheul-Ro Lee, Yong-Ho Ra

**Affiliations:** Division of Advanced Materials Engineering, Engineering College, Research Center for Advanced Materials Development (RCAMD), Jeonbuk National University, Jeonju, 54896 Republic of Korea

**Keywords:** Green energy system, Hydrogen evolution, CO_2_ reduction, III–V semiconductors, Photo electrochemical water splitting

## Abstract

Long-term societal prosperity depends on addressing the world’s energy and environmental problems, and photocatalysis has emerged as a viable remedy. Improving the efficiency of photocatalytic processes is fundamentally achieved by optimizing the effective utilization of solar energy and enhancing the efficient separation of photogenerated charges. It has been demonstrated that the fabrication of III–V semiconductor-based photocatalysts is effective in increasing solar light absorption, long-term stability, large-scale production and promoting charge transfer. This focused review explores on the current developments in III–V semiconductor materials for solar-powered photocatalytic systems. The review explores on various subjects, including the advancement of III–V semiconductors, photocatalytic mechanisms, and their uses in H_2_ conversion, CO_2_ reduction, environmental remediation, and photocatalytic oxidation and reduction reactions. In order to design heterostructures, the review delves into basic concepts including solar light absorption and effective charge separation. It also highlights significant advancements in green energy systems for water splitting, emphasizing the significance of establishing eco-friendly systems for CO_2_ reduction and hydrogen production. The main purpose is to produce hydrogen through sustainable and ecologically friendly energy conversion. The review intends to foster the development of greener and more sustainable energy source by encouraging researchers and developers to focus on practical applications and advancements in solar-powered photocatalysis.

## Highlights

- In-depth review assesses III–V materials for efficient hydrogen generation and CO_2_ reduction in renewable energy technologies.
- Exploration of strategies for broad light absorption and increased efficiency in water splitting processes and CO_2_ reduction.
- Innovative electrode designs conclude the path for stable, large-scale implementation of clean energy systems.

## Graphical abstract

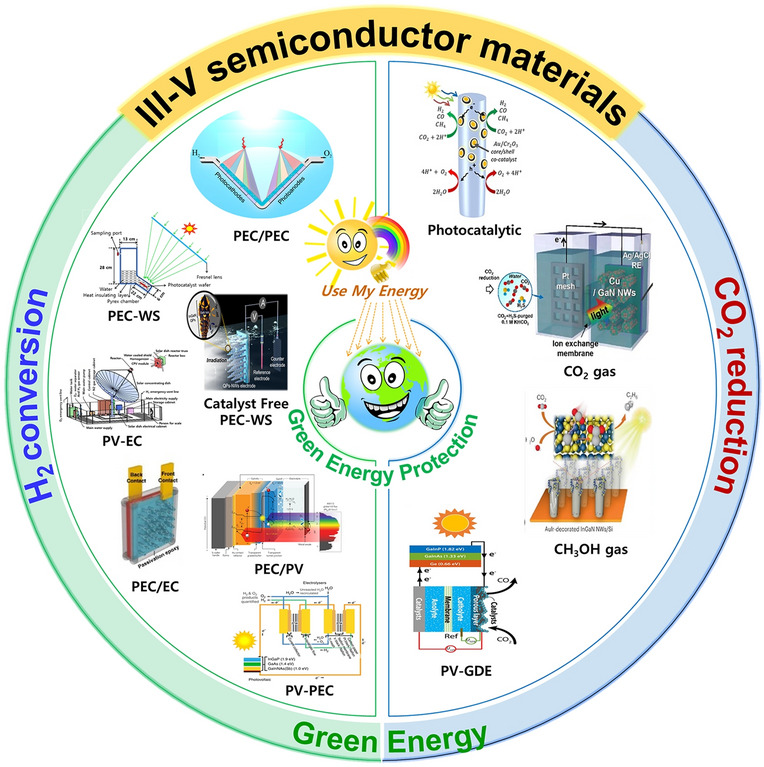

## Introduction

In recent decades, there has been a notable surge in global energy consumption, driven by significant technological advancements and a growing global population [1–6]. Fossil fuels are still the world’s main source of energy at the moment. The widespread use of fossil fuels has given rise to severe environmental problems [3, 7–11]. Solar energy, being a renewable source, offers a promising solution, and photocatalysis is a reliable method to harness solar energy and convert it into chemical energy [12–16]. Specifically, well-designed artificial semiconductor photocatalysts, when exposed to solar light, can split water into hydrogen (H_2_), which serves as an energy storage product [17–21]. Furthermore, a lot of study is being done on carbon dioxide (CO_2_) recycling as a potential energy technique. Catalytic reactions can reduce CO_2_, a significant contributor to global warming, into useful compounds [22–24].

In recent times, researchers have engineered artificial photosynthetic devices inspired by the natural photosynthesis process. These devices leverage solar energy to transform CO_2_ reduction and water into hydrocarbons [25–30]. The primary goal of these devices is to harness solar energy for the production of H_2_ fuel or carbon-based chemicals, utilizing CO_2_ and water in the process [31–33]. To achieve this, semiconductor materials are integrated into these devices. Light absorption by these semiconductors leads to the excitation of electrons into the conduction band and holes into the valence band [34, 35]. After that, the charge carriers produced by photosynthesis separate and proceed to the cocatalyst’s surface, where they actively participate in redox reactions [36–38]. However, low catalytic activity, ineffective charge carrier separation and transfer, and limited light absorption must be solved technologically before artificial photosynthetic devices are able to be widely used.

Three main categories comprise the current landscape of solar-driven CO_2_ reduction and H_2_ production technologies: electrochemical-photovoltaic (EC-PV), photovoltaic-electrochemical (PV-EC), and photoelectrochemical (PEC) water splitting. Despite the wealth of assessments in the domain of solar H_2_ production and CO_2_ reductions, the predominant emphasis is often placed on particular methodologies or strategies. This means that a substantial portion of the existing evaluations tends to concentrate on specific techniques or approaches within the broader scope of solar-driven H_2_ production and CO_2_ reduction, potentially leaving other potential avenues or methods less explored or underrepresented in the literature. A thorough examination of III–V semiconductor-based solar energy applications for CO_2_ reduction and H_2_ generation, considering long-term stability, high efficiency, and technical and economic viewpoints, has not been found in many reviews to date. A thorough analysis of this kind is essential to provide illuminating perspectives that clear the path for the future creation of highly efficient and commercially feasible solar-powered H_2_ production and CO_2_ reduction systems.

### Photoelectrochemical Water Splitting

Various semiconductors have been explored for photoelectrodes in PEC water splitting, including metal oxides, nitrides, and sulfides [39, 40]. Metal oxides, with their wide band gaps and unsuitable conduction band edges, often exhibit limitations such as low carrier mobility and poor stability, resulting in low photocurrent density [41–43]. III-nitrides, particularly InGaN, have gained attention due to excellent catalytic activity, electrical and optical properties, and stability. InGaN’s tunable bandgap across the solar spectrum positions it as an efficient and stable photoelectrode [44–47]. The InGaN/Si double-junction photocathode achieved a record-high solar-to-hydrogen efficiency of approximately 10.3% [48]. Notably, in 2023, Mi et al. set a world record efficiency of 9% for InGaN-based electrodes in photoelectrochemical water splitting using seawater as the electrolyte [49]. The material’s high chemical stability and catalytic activity make 1D III-nitrides promising for solar water splitting and CO_2_ reduction reactions (Tables 1, 4).

**Table 1:** Summary of III-nitride photocatalytic for overall water splitting using natural electrolytes

Photocatalyst	Cocatalyst	Light source	Electrolytes	STH(%)	Refs.
InGaN/GaN nanowires	Rh/Cr_2_O_3_, Co_3_O_4_	Xe lamp with AM 1.5G filer	Seawater	9.2	[49]
InGaN/GaN nanowires	Rh/Cr_2_O_3_, Co_3_O_4_	Natural solar light	Seawater	6.2	[49]
P-InGaN/GaN NWs	Rh/Cr_2_O_3_	Xe lamp with AM 1.5G filer	Seawater	1.9	[50]
InGaN/GaN core/shell NWs	Rh/Cr_2_O	Xe lamp with AM 1.5G filer	H_2_O	5.2	[51]
InGaN/GaN NWs	Rh/Cr_2_O and Co_3_O_4_	Xe lamp with AM 1.5G filer	H_2_O	2.7	[52]
P-GaN/InGaN NWs	Rh/Cr_2_O	Xe lamp with AM 1.5G filer	H_2_O	1.8	[53]
Mg-doped InGaN nanosheets	Rh/Cr_2_O	Xe lamp with AM 1.5G filer	H_2_O	3.3	[54]

**Table 2:** Summary of type of PV devices for solar water splitting

Type of PV devices	PV efficiency (%)	No of cells	Electrolytes	STH (%)	Refs.
InGaP/GaAs/GaInNAs (sb)	33.0	1	1.0 M H_2_SO_4_	16	[65]
GaAs/GaAs	16.3	1	1.0 M KOH	13.9	[66]
GaN/GaAs	15.3	1	1.0 M KOH	12.6	[66]
InGaP/GaAs(Ge)	31	1	1.0 M KOH	24.4	[67]
InGaAsP/GaAs	18.3	1	Kpi (pH7)	8.1	[67]
GaInP/GaAs	12	1	1.0 M KOH	16	[68]
GaInP/GaInAs/Ge	43	1	5.0 M KOH	28	[69]
GaInP/GaInAs(Ge)	44	6	Alkaline water	20	[70]
GaInP/GaInAS	33	6	Alkaline water	22	[71]
P-GaInP/GaAs	-	1	2.0 M KOH	16	[72]
Cu(In,Ga)Se2	17	3	3.0 M H_2_SO_4_	11	[73]
Crystalline-Si	16	4	0. 5 M Kbi (pH9.2)	10	[73]
Amorphous-Si	15	4	1.0 M KOH	9.5	[74]

### Photovoltaic-Electrochemical Water Splitting

Photovoltaic (PV)-based systems represent a rapidly advancing frontier in renewable energy technologies. However, to ensure a continuous power supply [55], these systems necessitate integration with additional energy storage and management solutions. Remarkably, PV-electrochemical (EC) devices enable water splitting even in the absence of light, utilizing the voltage generated by PV [56]. In PV-EC integrated devices, the inclusion of light-active catalytic electrodes reduces the required voltage output from PV. Single-crystalline devices, specifically those based on Si, InP, GaAs, and GaInP, exhibit the most favorable power conversion efficiency (PCE) and photovoltaic characteristics [57]. GaAs-based solar cells (SCs) play a pivotal role in enhancing hydrogen production rates in photoelectrolysis, offering a sustainable alternative to power supply electricity [58]. Remarkably, a single GaAs SC, operated at a voltage bias between 0.6 and 0.8 V, achieves efficient water splitting. Various strategies, including the adjustment of effective resistances, are employed to enhance current matching, bringing the operating points closer to the GaAs SC P_max_ point. The hybrid approach, integrating GaAs SC with a photoactive anode/cathode, emerges as a promising method for effective water splitting [59–62]. The gas generation rate of GaAs SCs is further amplified under intense light. A double-junction InGaP/GaAs-based photovoltaic device, with a low ηO_2_ value, holds promise for solar water splitting under 1 sun irradiation conditions [82]. Despite achieving a benchmark solar-to-hydrogen (STH) efficiency of 12.4%, the practical application of monolithic tandem devices is hindered by challenges such as high costs and short lifespans [64]. Notably, a device without a photoelectrode, known as a buried PV cell, can still be termed a PEC cell, achieving a maximum STH efficiency of 30% with the integration of two polymer electrolyte membrane electrolyzers and an InGaP/GaAs/GaInNAs(Sb) triple-junction solar cell [63]. However, challenges, including expensive material costs, remain to be addressed (Table 2).

### Photoelectrochemical-Photovoltaic Water Splitting

Photoelectrochemical-photovoltaic (PEC-PV) systems stand out as simple and cost-effective means for H_2_ production [75], dispersing the photocatalyst power of semiconductors in pure water under solar irradiation. While certain PC systems have achieved a maximum STH efficiency of 5%, challenges persist in enhancing either the solar-to-STH ratio or stability [76]. PEC systems, incorporating photoanodes and photocathodes in an electrolyte solution, have demonstrated significantly higher PV values, reaching a maximum of 47% [77]. Both PC and PEC systems necessitate the development of innovative photoactive materials capable of capturing longer-wavelength solar light and efficient cocatalysts to enhance H_2_ evolution reactions and improve STH in solar water splitting [37]. Photoelectrochemical-Photovoltaic (PEC-PV) systems, connecting photovoltaic devices with electrolyzers, offer a promising avenue benefiting from advancements in both PV devices and electrolytic water splitting [38]. PV devices must exceed the theoretical thermodynamic potential plus overpotential for water splitting, often necessitating multi-junction PV devices or multi-connected cells in series [78]. Various PV devices, including organic, perovskite, silicon, and compound semiconductors, have been employed, achieving STH values ranging from 6.1% to 16% at 1 sun irradiation conditions. A triple-junction InGaP/GaAs/GaInNAs(Sb) PV device, with 2.8 V of open-circuit voltage, attained 16% STH at 1 sun irradiation conditions when connected to an electrolyzer [118]. The review’s focus is the various water splitting methods for hydrogen generation and CO_2_ reduction, we then discuss in detail the different solar water splitting technologies and their underlying concepts, as well as the solar-drive CO_2_ reduction systems (Table 3).

**Table 3:** Summary of PEC-PV devices for solar water splitting

Photoelectrochemical-photovoltaic (PEC-PV)	Light source	Electrolytes	STH (%)	Refs.
GaN/GaInP_2_/GaAs(Ge)	Am 1.5G (1 sun illumination)	0.1 M H_2_SO_4_	12	[79]
NiMo/GaAs/ InGaP/TiO_2_/Ni	Am 1.5G (1 sun illumination)	0.1 M KOH	8.6	[80]
GaAs/GaInAs/GaInP/GaInAs(Ge)	Am 1.5G (1 sun illumination)	1.0 M HClO_4_	14	[81]
RuO_2_-GaAs/GaInAs/GaInP/AlInp	Am 1.5G (1 sun illumination)	1.0 M HClO_4_	19	[82]
TiO_2_-InGaNP/GaAs(Ge)	Am 1.5G (1 sun illumination)	3.0 M KHCO_3_	11.2	[83]
P-GaAs/n-GaAs/P-GaInP_2_	White ilght illumination	3.0 M H_2_SO_4_	12.4	[84]
MoS_2_-GaInAs/GaAs	Am 1.5G (1 sun illumination)	0.5 M H_2_SO_4_	6.1	[85]
PtRu-pn-GaInAsP/pn-GaAs	Am 1.5G (1 sun illumination)	0.5 M H_2_SO_4_	7.6	[86]
InGaP/GaAs	Am 1.5G (1 sun illumination)	1.0 M Na_2_SO_4_	9	[87]
GaInP/GaAs	Am 1.5G (1 sun illumination)	0.5 M H_2_SO_4_	13.6	[88]

**Table 4:** Summary of III-nitride photocathode for solar CO_2_ reduction

Photocathode	Cocatalyst	Light source	Electrolytes	Main products	Current density (mA cm^−2^)	Refs.
GaN/Si	Cu-Fe	1 sun illumination	Pure CO_2_	CH_4_	38.3	[172]
GaN/Si	Cu	–	Pure CO_2_	CH_4_	30	[173]
GaN/Si	TiO_2_-Pt	8 sun illumination	Pure CO_2_	CO, H_2_	15	[174]
GaN/Si	Sn	1 sun illumination	Pure CO_2_	HCOOH	11	[175]
GaN/Si	Au	1 sun illumination	Pure CO_2_	CO, H_2_	3	[176]
GaN/Si	Cu-Fe	1 sun illumination	CO_2_, H_2_S	HCOOH	7.07	[177]
GaN/Si	AuPt	1 sun illumination	Pure CO_2_	CO, H_2_	9	[178]
GaN/Si	Au	1 sun illumination	Pure CO_2_	CO	1	[179]
p-GaN films	Au	1 sun illumination	Pure CO_2_	CO	0.0013	[180]

### Basic Principles of CO_2_ Reduction and H_2_ Conversion

#### PEC in CO_2_ Reduction and H_2_ Conversion

The fundamental principles of PEC water splitting and CO_2_ reduction involve addressing the thermodynamic requirements, as illustrated in the process of splitting water molecules into hydrogen and oxygen, as per Eq. 1. The free energy change for this process under standard conditions is Δ*G* = 237.2 kJ mol^−1^. The reversible potential difference for this reaction can be determined by the Nernst equation: *E*° = 1.23 V per electron transported. Notably, the overall water splitting reaction comprises two half-cell reactions known as the hydrogen evolution reaction (HER) and the oxygen evolution reaction (OER) [28, 89]:

1 $${\text{H}}_{{2}} {\text{O}} + { 2}\left( {{\text{h}}^{ + } } \right) \to {1}/{\text{2 O}}_{{2}} + {\text{ 2H}}^{ + } \,\left( {{\text{OER}}} \right) {\text{E}}^\circ_{{{\text{OER}}}} = \, - {1}.{\text{23 V vs NHE}}$$

2 $${\text{2 H}}^{ + } + {\text{ 2e}}^{ - } \to {\text{H}}_{{2}} \,\left( {{\text{HER}}} \right) {\text{E}}^\circ_{{{\text{HER}}}} = \, 0.00{\text{ V vs NHE}}$$

3 $${\text{H}}_{{2}} {\text{O}} \to {\text{H}}_{{2}} + { 1}/{\text{2 O}}_{{2}} \Delta \,{\text{E}}^\circ \, = \, - {1}.{\text{23V}}$$

**Fig. 1:**
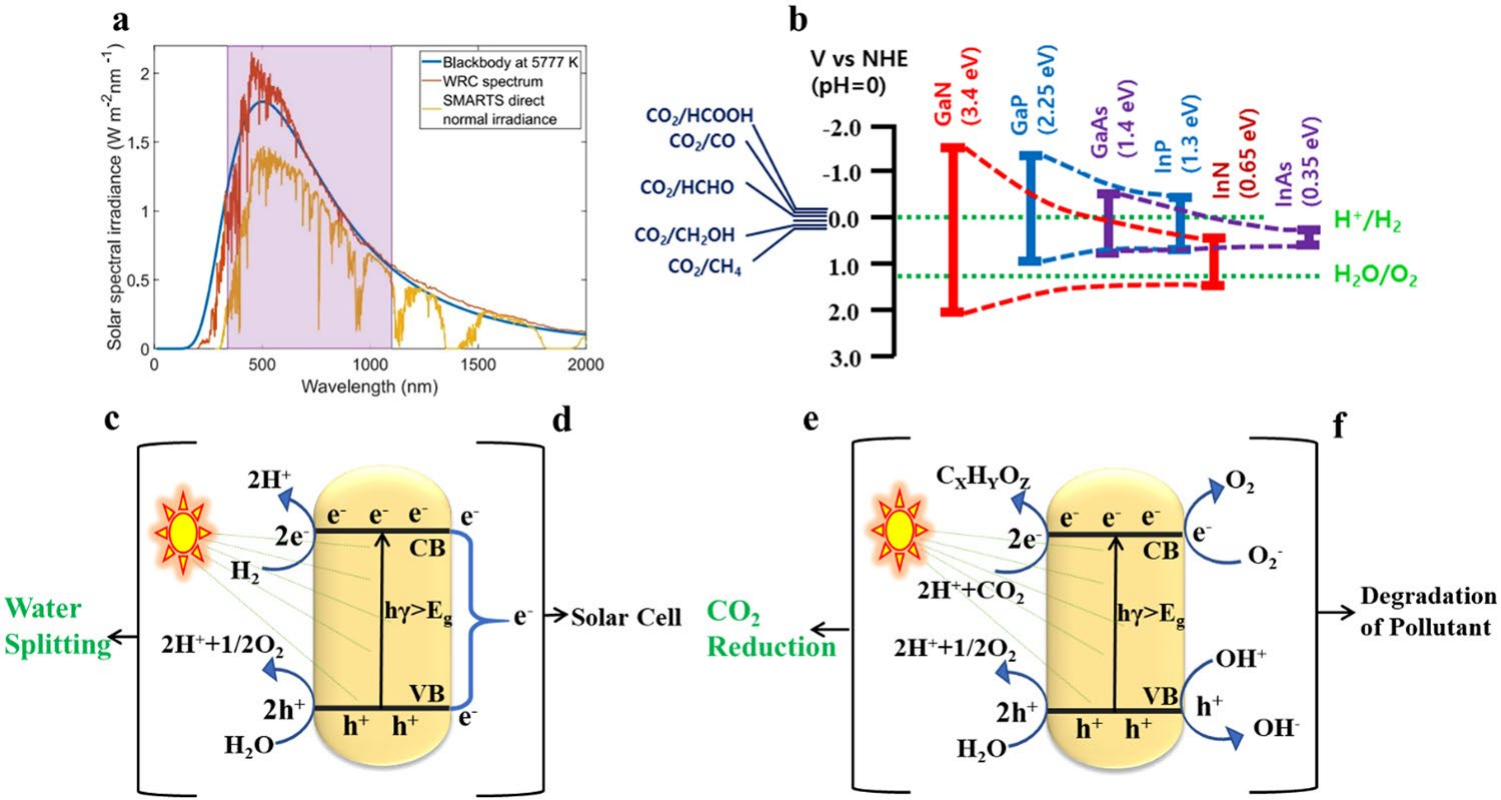
Illustrates various aspects of solar spectrum utilization and semiconductor photocatalysis. In **a**, the solar spectrum at the Earth’s surface is depicted. Reproduced with permission from Ref. [181]. Copyright 2022 Springer Scientific Reports, **b** provides a schematic highlighting the tunable band structure in contrast to traditional semiconductors [182]. The mechanisms of photocatalytic semiconductors are detailed in **c-f**: **c** water splitting Reproduced with permission from Ref. [183]. **d** Solar cell applications [119]. **e** CO_2_ reduction [184]. and **f** pollution degradation [184]

For every half reaction, the standard electrode potential (E°) measured at pH 0 and room temperature can be converted to the electrochemical potential, denoted by -*q*E°, where *q* is the elementary charge. In order for a PEC (photoelectrochemical) cell reaction to happen on its own, the semiconductor’s conduction band must be placed at a more negative potential than E°_*H*ER_ (hydrogen evolution reaction) and the valence band at a more positive potential than E°_*O*ER_ (oxygen evolution reaction) (Fig. 1c).

In order to facilitate the production of different products by providing protons (H^+^) to the photocathode, the water oxidation reaction is necessary in order to permit the reduction of CO_2_ inside a PEC cell. The normal hydrogen electrode (NHE) at pH 7 under room temperature and pressure circumstances is the reference point for the thermodynamic potential for the reduction of CO_2_ into different products (Fig. 1e) in Eq. 2 [90, 91]:

$${\text{2H}}^{ + } + {\text{ 2 e}}^{ - } \to {\text{H}}_{{2}}\, {\text{E }} = \, - 0.{\text{41V}}$$

$${\text{CO}}_{{2}} + {\text{ e}}^{ - } \to {\text{CO}}_{{2}}^{ - } \,{\text{E }} = \, - {1}.{\text{85V}}$$

$${\text{CO}}_{{2}} + {\text{ 2 H}}^{ + } + {\text{ 2 e}}^{ - } \to {\text{CO }} + {\text{ H}}_{{2}} {\text{O}} \,{\text{E }} = \, - 0.{\text{52V}}$$

$${\text{CO}}_{{2}} + {\text{ 2 H}}^{ + } + {\text{ 2 e}}^{ - } \to {\text{H CO}}_{{2}} {\text{H}} \,{\text{E }} = \, - 0.{\text{61 V}}$$

$${\text{CO}}_{{2}} + {\text{ 4 H}}^{ + } + {\text{ 4 e}}^{ - } \to {\text{H COH }} + {\text{ H}}_{{2}} {\text{O}} \,{\text{E}} = \, - 0.{\text{48 V}}$$

$${\text{CO}}_{{2}} + {\text{ 6 H}}^{ + } + {\text{ 6 e}}^{ - } \to {\text{CH}}_{{3}} {\text{OH }} + {\text{ H}}_{{2}} {\text{O}} \,{\text{E }} = \, - 0.{\text{39 V}}$$

$${\text{CO}}_{{2}} + {\text{ 8 H}}^{ + } + {\text{ 8 e}}^{ - } \to {\text{CH}}_{{4}} + {\text{ 2H}}_{{2}} {\text{O}} {\text{E }} = \, - 0.{\text{24 V}}$$

These reduction potentials demonstrate the strong thermodynamic correlation between the reduction of carbon dioxide and the reduction of protons to produce hydrogen. As a result, it becomes extremely important for the photoelectrocatalyst employed in this process to be precise [168]. It is essential to note that the distinction between the standard reduction potential and the reduction potential for proton to hydrogen at pH 7 is elucidated by the Nernst equation, where V = E° − 0.059*x*. Here, the variable *x* represents the pH of the solution.

In the CO_2_ reduction reaction, the band alignment mirrors that of PEC water splitting. In both cases, the valence band of a photoelectrode must be positioned higher than the potential for oxidation reaction, while its conduction band should be lower than the potential for reduction reaction. The excitation of an electron–hole pair in the semiconductor requires photon energy above the bandgap. To enhance photocurrent density in a photovoltaic cell, it is crucial to effectively separate these electron–hole pairs before they undergo recombination [92, 93].

PEC devices show an intrinsic electric field at the junction between the semiconductor-liquid Junction (SCLJ). This is in contrast to photovoltaic devices, which rely on a p–n junction to facilitate the separation of electron–hole pairs [94]. The electric field at the semiconductor-liquid interface creates a space charge zone within the semiconductor surface, generated by the equilibrium of the electrochemical potential (Fermi level) between the semiconductor and the relevant redox couplings in the solution [95, 96]. As seen in Fig. 1e, this space charge area also bends the semiconductor’s electronic band. Numerous research efforts have been directed at improving charge separation efficiency through techniques such as nanostructuring [97], depositing overlayer films [98], forming p-n junctions [99], and introducing dopants into the semiconductor. This article does not go into detail on the complex mechanics of the semiconductor-liquid interface.

An integral aspect of PEC cell research involves displacing the charge carriers from the surface of the semiconductor to facilitate the necessary reactions in the solution [100]. Consequently, it is customary to employ various modifications to enhance the performance of semiconductor electrodes in hydrogen production and CO_2_ reduction. These modifications may include the addition of dopants and cocatalysts, as well as the construction of underlayers or overlayers [36, 101–104]. In addition, the semiconductor materials utilized in PEC cells need to be easily available, affordable, and ecologically harmless, as well as have long-term stability in aquatic environments [105–108].

The ideal (Schottky-Queisser) solar energy conversion efficiency of approximately 34% places a limit on photocatalytic water splitting with a single absorber and two photons. After accounting for reasonable losses, it can actually reach a maximum possible efficiency of about 10%. Achieving devices with high efficiency has proven to be exceptionally challenging due to the scarcity of suitable photocatalytic materials possessing the appropriate bandgap, optimal conduction and valence band levels, and stability under rigorous photocatalytic conditions [109].

Metal oxides have long been the subject of much research as photocatalysts for water splitting and CO_2_ reduction; however, because of their wide bandgaps and low activity under visible light irradiation [110, 111], which accounts for around 46% of solar spectrum energy, their efficiency is just 3% [112, 113]. As previously mentioned, visible light-responsive photocatalysts frequently exhibit instability and oxidation tendencies [105–108], which limits their efficacy in the visible spectrum.

Moreover, due to their suboptimal crystalline quality, restricted surface-to-volume ratio, and inefficient light absorption and charge carrier separation, photoelectrodes made of metal oxides frequently exhibit less-than-ideal opto-electronic and catalytic properties [48, 114, 115]. Therefore, to enable practical large-scale hydrogen production and CO_2_ reduction, it becomes imperative to explore innovative photocatalytic devices that are not only stable but also efficient under visible light irradiation.

Semiconductors belonging to the III–V compound group, in particular, demonstrate remarkable stability under photocatalytic conditions and feature a direct energy bandgap that covers a substantial portion of the solar spectrum, as illustrated in Fig. 1a. The exact construction and design of these III–V compound semiconductors enable their application in a range of photo(electro)chemical systems. Ternary alloying in III–V compounds enables precise adjustment of the band gap and lattice parameters. This method promotes the epitaxial growth of high-quality crystals and facilitates the accurate alignment of band boundaries. Because of their adaptability, III–V semiconductors can support a wide range of redox potentials and span the entire solar spectrum with fine control over the energy band gap (Fig. 1b).

Moreover, they prove adaptable for applications in H_2_ conversion, solar cells, CO_2_ reduction, and pollutant degradation due to their adjustable bandgap that accommodates a significant portion of the solar spectrum, including ultraviolet, visible, and near-infrared regions [97, 116, 117]. The heightened photocatalytic activity of III–V compound semiconductor devices can be attributed to their high surface-to-volume ratio and effective separation of charge carriers.

Understanding how different forms of light (UV, visible, and infrared) affect the performance of different semiconductors is fundamental for understanding the principles of photocatalysis. The range of light absorption is a significant component influencing the photocatalytic activity of a semiconductor.

The band theory of solids elucidates the photocatalytic process within a single-component semiconductor (Fig. 1c–f). This process entails the movement of electrons from the valence band (VB) to the conduction band (CB), triggered by the generation of holes in the VB due to the absorption of incident photons [119]. Upon reaching the semiconductor’s surface through diffusion, a portion of these charges gets absorbed by various entities. For instance, in the degradation of pollutants, oxygen can absorb electrons to generate superoxide radicals [120]. In the process of water splitting, electrons may combine with hydrogen ions to form hydrogen gas. Unfortunately, a significant fraction of the remaining charges tends to recombine, which is detrimental to the overall photocatalytic activity.

Photocatalysis manifests in various forms depending on the application. In water oxidation sites, holes are taken up by water to create H^+^ and O_2_, and subsequently, H^+^ combines with electrons to produce H_2_, a process known as water splitting [121]. Within a solar cell, the recombination of electrons with holes gives rise to the generation of an electric current. In CO_2_ reduction, holes are captured by water, leading to the formation of H^+^ and O_2_. These products then react with CO_2_ in the presence of electrons, resulting in the production of C_x_H_y_O_z_. This specific process is commonly known as CO_2_ reduction [122]. The degradation of contaminants leads to the release of electrons and holes, with dissolved oxygen and water capturing them, respectively. Consequently, reactive oxygen species such as superoxide (⋅O¬2 –) and hydroxyl radicals (·OH) are generated, rapidly breaking down various organic pollutants into CO_2_ and H_2_O) (Fig. 1c–f).

#### PV-EC in CO_2_ Reduction and H_2_ Conversion

The PV cell and the electrolyzer are the two separate parts of the PV-EC system (Fig. 3b) and (Fig. 12b). The EC cell is directly powered by the electricity produced by the photovoltaic cell’s absorption of solar radiation to split water into H_2_ conversion and CO_2_ reduction at the cathode and anode. To avoid corrosion caused by water, the PV cell is attached to the cathode and anode but stays apart from the pure CO_2_ gas and water electrolyte. An important benefit of PV-EC water splitting is the commercial availability and technological maturity of both water electrolysis and PV cells. High solar-to-H_2_ efficiency and accessible CO_2_ reduction are made possible by commercial PV cells and electrolyzers with efficiencies above 18% and 60–83%, respectively [123–126].

#### PEC-PV in H_2_ Conversion

The system immerses itself in electrolyte for water splitting in the semi-integrated PV/PEC water splitting arrangement, which includes both a hidden junction and a semiconductor/electrolyte junction (Fig. 8a). Some devices in this category combine semiconductor photoelectrodes with photovoltaic modules to act as light absorbers; these are called photoelectrode-modified PV cells. For real-world applications, these designs are meant to maximize device performance by maximizing conversion efficiency and minimizing losses. Integrated devices using Gallium arsenide-based solar cells usually have electrodes modified from PV modules; anode electrocatalysts are deposited directly on the PV side and coupled to the cathode [127, 128].

## Recent Advances in Solar Hydrogen Production

### Photocatalytic Water Splitting

In photoelectrochemical water splitting, O_2_ evolution at the photoanode and H_2_ evolution at the photocathode are the two main half-reactions. An applied cathodic potential drives photogenerated electrons in semiconductors during the photoelectrochemical H_2_ evolution reaction. When light with energy exceeding the band gap of the semiconductor is absorbed, photogenerated holes persist in the valence band, concurrently elevating electrons to the conduction band. These conduction band photogenerated electrons move to the electrode/electrolyte interface at the surface, where they take part in the H_2_ evolution reaction [28, 88]. Therefore, for an effective H_2_ evolution reaction, the photocathode’s conduction band edge should be positioned above water’s reduction potential. Conversely, as seen in the photoelectrochemical O_2_ evolution reaction, an anodic potential allows the photogenerated holes in semiconductors’ valence band to participate in the water oxidation reaction. As a result, the photoanode’s valence band edge needs to be below water’s oxidation potential [28, 88].

**Fig. 2:**
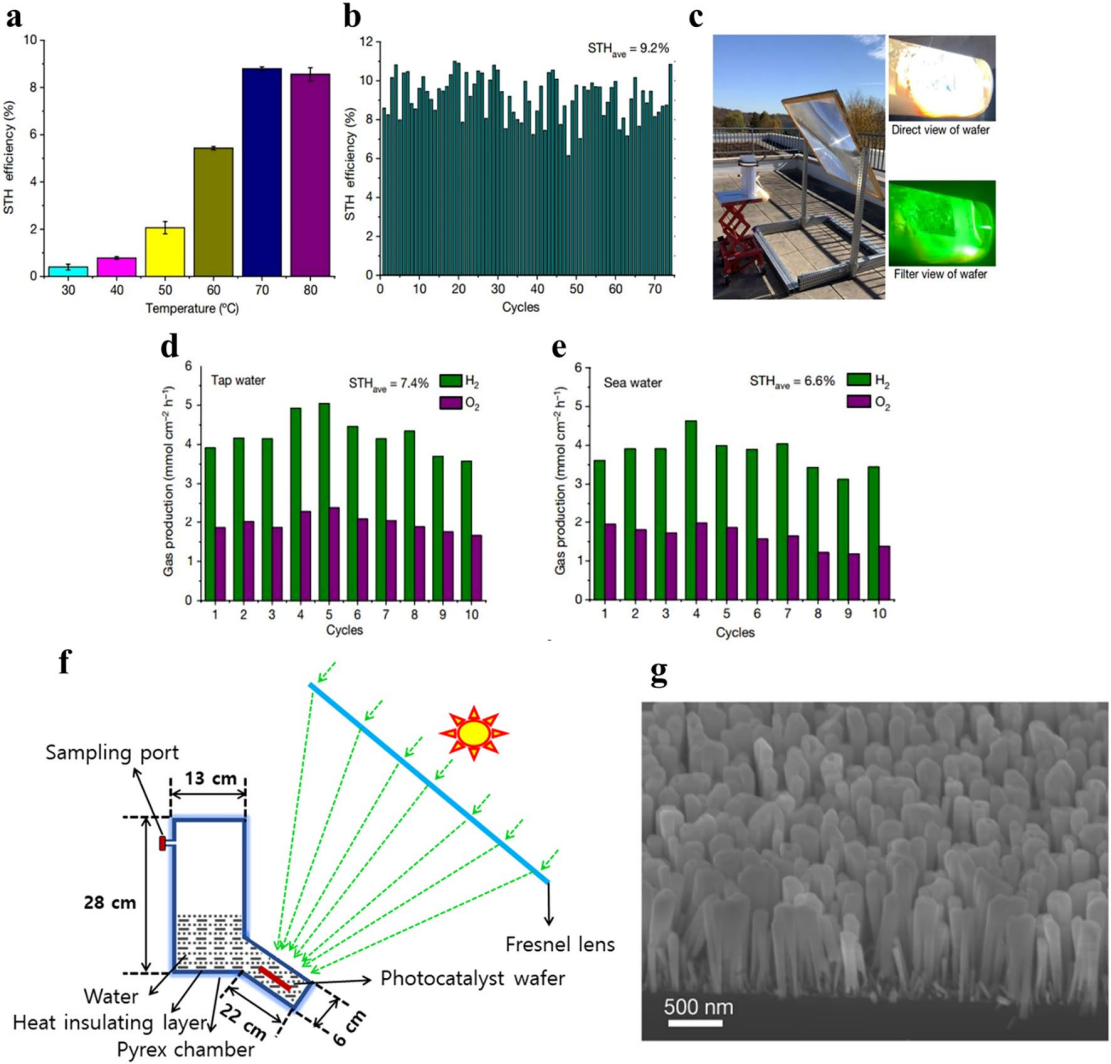
**a** Photocatalytic overall water splitting (OWS) system demonstrating ultra-high solar-to-hydrogen (STH) efficiency. **b** Depiction of the temperature-dependent STH efficiency of InGaN/GaN nanowires and cocatalyst Rh/Cr_2_O_3_/Co_3_O_4_-nanowires under a light intensity of 3800 Mw cm^−2^. **c** Outdoor photograph capturing the setup of the photocatalytic OWS system. **d** Indoor measurements of STH in tap water under a light intensity of 3800 mW cm^−2^, recorded in hourly intervals. **e** Outdoor measurements of STH in sea water under a light intensity of 16,070 mW cm^−2^, taken in 10-min intervals. **f** Schematic parameter illustration detailing the outdoor photocatalytic OWS setup. Reproduced with permission from Ref. [49]. Copyright 2023 Springer Nature

The conduction and valence band edge positions required for water reduction and oxidation are present in materials like III-nitrides, such as GaN or InGaN [129]. As such, they are great substitutes for examining the characteristics of photoelectrochemical water splitting. More specifically, InGaN/GaN nanostructures—that is, nanorods, nanowires, and nanowalls—have garnered attention due to their higher photoelectrochemical water splitting activity in comparison with planar structures [130]. InGaN/GaN nanowires coated with Rh/Cr_2_O_3_/Co_3_O_4_ cocatalysts, a remarkable increase in STH efficiency was observed upon temperature rise (Fig. 2a). At 70 °C, the STH efficiency reached its highest point of 8.8%. With the exception of the water desorption stage in H_2_-O_2_ recombination, which indicates that H_2_-O_2_ recombination is suppressed as temperature rises, theoretical simulations showed that the majority of the steps in the water splitting reactions are exothermic. However, increasing the ambient temperature to 80 °C did not enhance the STH efficiency because the higher diffusivity of H_2_ and O_2_ in water promoted H_2_–O_2_ recombination.

With a STH efficiency of 9.2%, the Rh/Cr_2_O_3_/Co_3_O_4_-InGaN/GaN photocatalyst set the world record. It also showed encouraging stability over 74 h at the optimal temperature of 70 °C (Fig. 2b). An even larger and simpler water splitting apparatus was used, with a 4 cm × 4 cm photocatalyst wafer subjected to intense solar light (about 16,070 mW cm^−2^), producing 257 W. Experiments conducted outside showed that the photocatalyst wafer remained stable under the highest intensity of concentrated solar light employed in photocatalytic overall water splitting, and that an insulating layer-coated chamber maintained the ideal operating temperature of 75 ± 3 °C (Fig. 2c). During tests carried out in tap water and saltwater (Fig. 2d, e), the photocatalysts showed outstanding STH efficiencies of 6.6% and 7.4%, respectively. This emphasizes how highly applicable they could be in real-world situations.

In theory, the capacity to absorb more solar energy in a particular irradiated area suggests the idea of perhaps lowering material prices. An investigation was conducted by submerging a 4 cm by 4 cm photocatalyst film in water and exposing it to high sunlight (about 16,070 mW cm^−2^). During outside testing, the experimental setup proved that it could self-heat and maintain an elevated temp. of 75 ± 3 °C. 6.2% was the highest STH efficiency ever measured in ambient light (Fig. 2e). Furthermore, a schematic depiction of the outdoor photocatalytic overall water splitting (OWS) system’s parameters is shown in Fig. 2f. A roughly 50 cc device had a 4 cm × 4 cm photocatalyst film that was subjected to prolonged outdoor testing in sunlight directly. 300 ml of deionized water were contained in a 4,350-ml chamber in which the wafer and holder were properly placed. The 4 cm × 4 cm photocatalyst wafer was positioned over a region of about 8 cm × 8 cm, which was focused solar light (about 16,070 mW cm^−2^) using a 1.1 m × 1.1 m Fresnel lens (Fig. 2f). The results of this article show that hydrogen may be produced efficiently with direct sunlight and either tap water or sea water.

### PV-EC Water Splitting (Three-Junction Solar Cell)

**Fig. 3:**
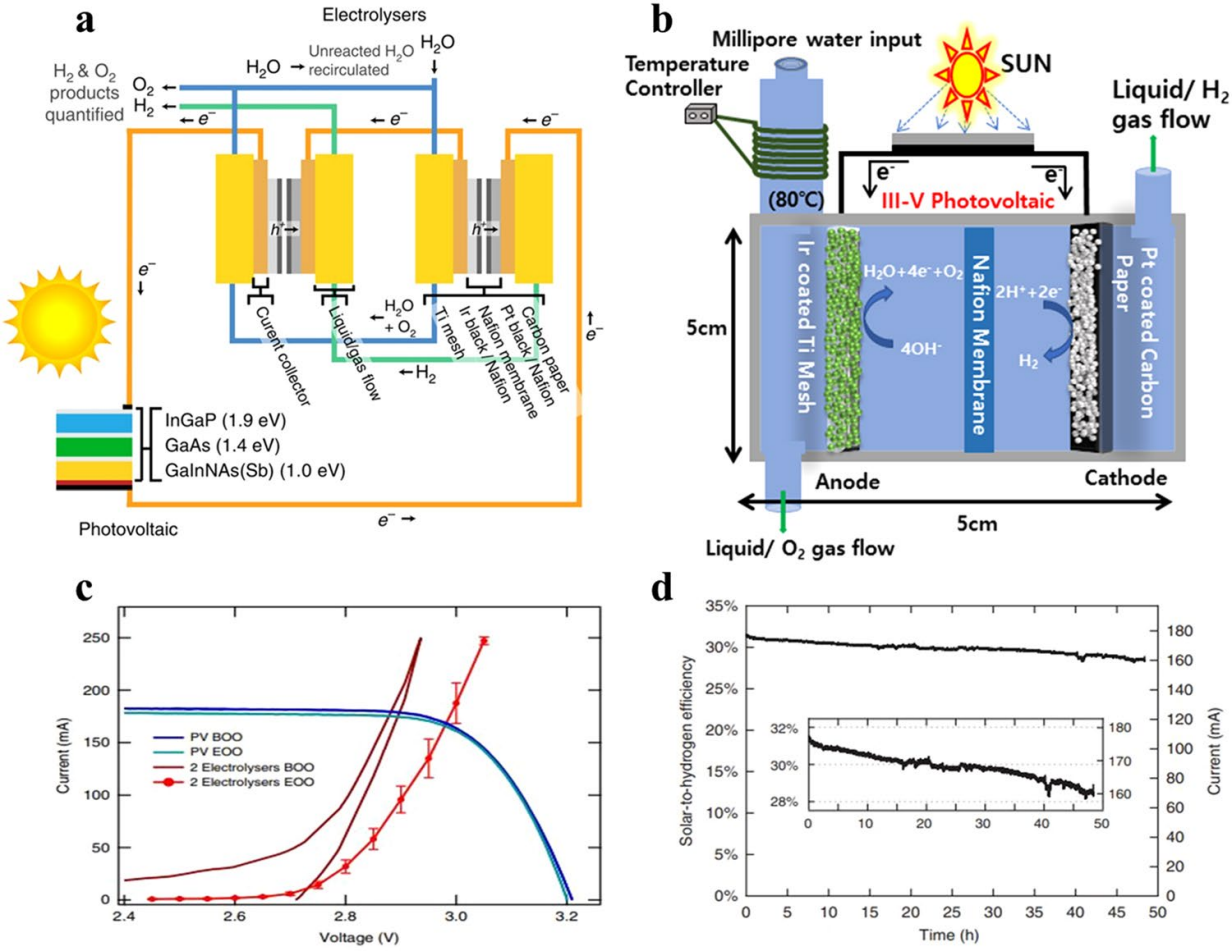
Photovoltaic-electrochemical (PV-EC) system configuration **a** an illustration of a photovoltaic-electrochemical (PV-EC) system is shown, consisting of two PEM electrolyzers coupled in series and a three-junction (GaInNAs(Sb)/GaAs/InGaP) solar cell. **b** Schematic illustration of parameters in the PV-EC electrolysis system. **c** The current–voltage (*I–V*) characteristics of the PV cell and performance of the PEM electrolyzer at the beginning and end of the operational period. **d** Efficiency of STH versus time for the PV-EC system. Reproduced with permission from Ref. [ 63 ]. Copyright 2016 Springer Nature Communications

The individual performances of the PV and PEC components of a photovoltaic-electrical (PV-EC) system determine its overall efficiency. Modules made of CdTe, Cu(In,Ga)(Se,S)_2_ (CIGS), monocrystalline Si, and multicrystalline Si are currently available for commercialization. Record efficiencies of 22.4%, 18.5%, 17.5%, and 18.6% are claimed by these modules, in that order. Si-based modules command a significant market share of over 90% because of their remarkable stability, dependability, and affordability. Interestingly, Si-based modules show less than 10% efficiency degradation over a 25-year period [131]. As of now, 12.4% is the maximum efficiency that can be achieved with a PEC water splitting system that has at least one semiconductor–liquid junction [132]. Commercial PV cells still have opportunity for incremental efficiency improvements, but the Shockley–Queisser (S-Q) limit places a limit on the maximum theoretical efficiency of 33.7% for a semiconductor with a bandgap of 1.34 eV in single-junction solar cells [93]. It is possible for multi-junction solar cells to outperform the S-Q limit. A five-junction tandem arrangement of GaInAs/GaInP/GaAs/AlGaInAs/AlGaInP produced a record efficiency of 38.8%, although at significant manufacturing costs [65]. These systems are more costly because they require expensive membranes and noble metals like Pt, Ir, and Ru, but they also resolve some of the limitations of alkaline electrolyzers. Comparatively, because of their flexibility, PEM systems are better suited for integration with PV systems [123, 124, 133]. The significant discrepancy in STH efficiency reported for photovoltaic (PV) electrolysis devices and standalone solar-to-electricity PV systems is mainly caused by the IV characteristics of multi-junction photovoltaics and electrolyzers (water based) not being aligned properly [134]. One major barrier is that, in light circumstances ranging from 1 to 1,000 suns, the maximum power-point voltage (VMPP) of a typical commercial triple-junction solar cell falls between 2.0 and 3.5 V. On the other hand, the operational voltages in practical scenarios typically fall within the range of 1.5–1.9 V, but the thermodynamic minimum voltage required for water electrolysis is only 1.23 V at 300 K [135–138]. During water electrolysis, when the voltage surpasses the thermodynamic minimum, the excess energy is dissipated as heat rather than being stored in the chemical bonds of H_2_. This constraint was traditionally handled by connecting additional PV and/or electrolyzer units in series to optimize the voltage match between these device components. A schematic illustration of the PV-electrolysis system is shown in Fig. 1a. In this configuration, a commercially available triple-junction solar cell with an active area of 0.316 cm^2^ was used. To replicate concentrated AM 1.5D solar lighting, the solar cell was put on a water-cooled stage with a cell temperature of roughly 25 °C and irradiated with white light from a xenon arc lamp. The parameters of the PV-EC electrolysis system are schematically illustrated in Fig. 1b. The setup comprised two proton exchange membrane (PEM) electrolyzers, where Nafion membranes were coated with Pt black catalyst (cathode) and Ir black catalyst (anode). This PV cell, along with a potentiostat that measured current, was linked in series with these electrolyzers. In the first electrolyzer, water was fed into the anode compartment, while the cathode received no input flow. The water and O_2_ effluent from the first electrolyzer’s anode compartment poured into the second electrolyzer’s anode compartment. The H_2_ from the cathode side of the first electrolyzer flowed into the cathode side of the second electrolyzer at the same time. Any unreacted water was cycled back through the system while the produced H_2_ and O_2_ products were collected and measured in the second electrolyzer. The electrolyzers met the specifications for industrial water electrolyzers by operating continuously for 48 h at a temperature of about 80 °C. The results of the experiment revealed a maximum power-point solar-to-electricity efficiency of 39%, with a cell voltage (VMPP) output of 2.91 V and a current density (JMPP) of 565.9 mA cm^2^. Figure 3c shows the *I-V* characteristics of the dual electrolyzer and solar cell before and after the 48-h operation. The electrolysis current and associated STH efficiency for the full 48-h experiment is shown in Fig. 3d. Surprisingly, the operational current only fell by 10% throughout this time span, from 177 to 160 mA. With the highest average STH efficiency ever recorded, this PV-electrolysis system is the very first sunlight-powered water splitting system to achieve a STH efficiency of 30% or more. By connecting the multi-junction solar cell in series with two electrolyzers, the excessive voltage was successfully decreased, enhancing the utilization of the PV’s outstanding effectiveness for water splitting. Interestingly, the system used a lot of components that were readily accessible to purchase on the market, suggesting that it may be used extensively. With the successful demonstration of this high-efficiency system, the US Dept. of Energy’s technical and economical targets have been greatly exceeded, indicating intriguing future possibilities for solar water splitting-based hydrogen production and energy storage.

### PV-PEC Water Splitting (Tandem Junction Solar Cell)

For the widespread implementation and effective utilization of solar fuel technologies, it is crucial to conduct pilot-scale on-sun demonstrations to assess and showcase the feasibility of scaling up [89, 137–139]. Nevertheless, the scaling of PEC or integrated photovoltaic (PV) + electrolysis (EC) systems introduce numerous challenges depending on the specific device design, experimental setup, and materials employed [139]. Balancing competing requirements, including high efficiency, increased hydrogen production rates, stability for prolonged time, cost-effective, and environmentally sustainable, typically forms the crux of these challenges. Addressing these hurdles in scaling solar fuel systems is an intricate undertaking, but it can generally involve expanding the area of photo-absorber per device, and/or implementing solar concentration [140]. Notably, focused solar light has shown promise as a viable approach to achieving commercially feasible, high-power-density devices, allowing for the utilization of more expensive photo-absorber materials [141–144]. Among the many possible advantages of solar concentration is the ability to increase device efficiency [146] while also generating useful heat.

**Fig. 4:**
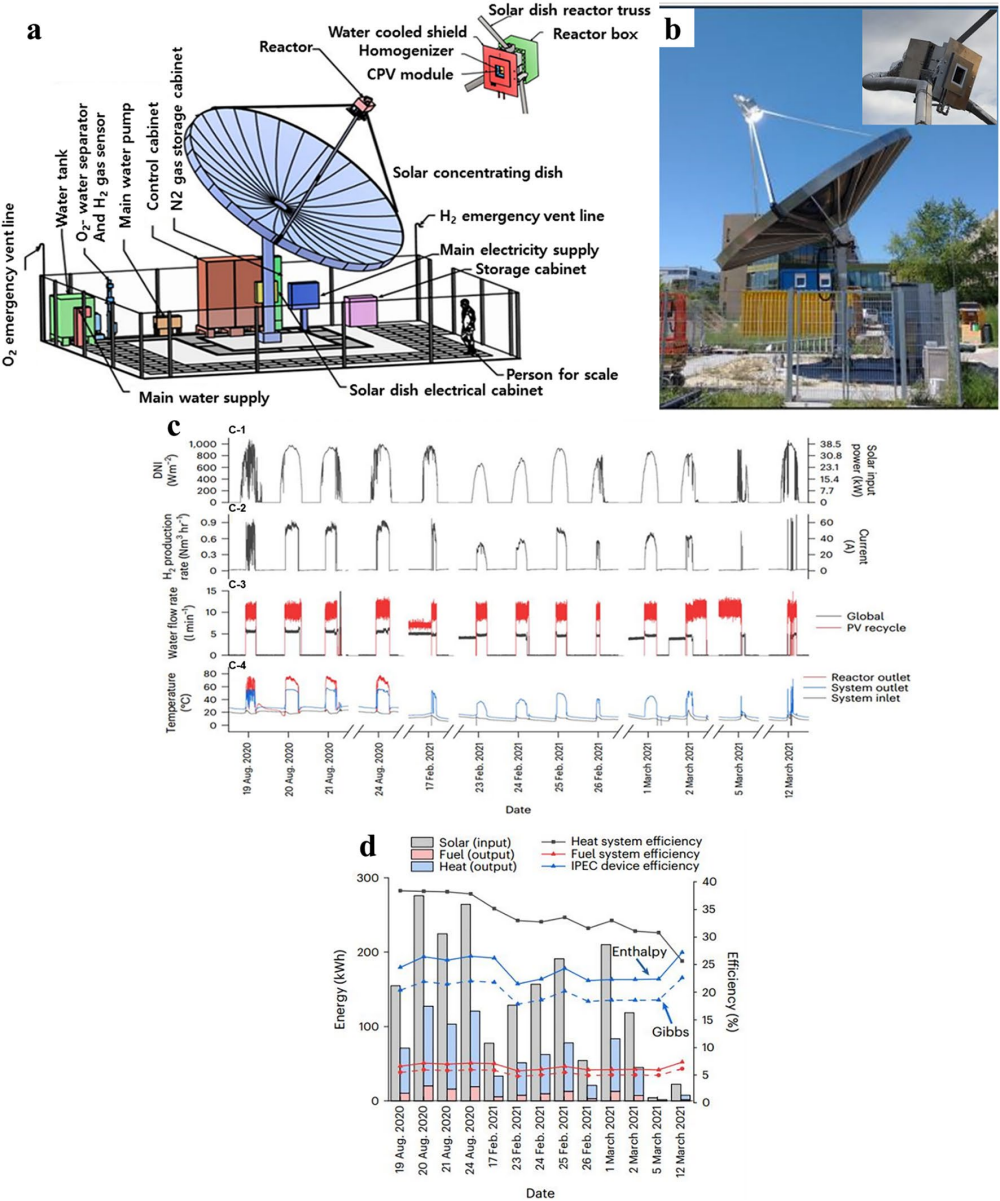
Dish system with solar parabolic concentrator. **a** A technical drawing that shows the layout of the complete site and includes the reactor, solar parabolic concentrator dish, and cabinet hardware. Photographs that display the reactor unit and the solar dish system **b**. The weather conditions varied during the operation times, as shown in **c**. **d** Also shows the immediate input and output powers that were measured during the course of the 13-day experiment. Reproduced with permission from Ref. [145]. Copyright 2022 Springer Nature Energy

The system configuration used in the study for the co-generation of heat, hydrogen, and oxygen from solar energy is shown in Fig. 4a, b. For the purpose of concentrating and directing concentrated solar energy into a solar reactor, a dual-axis tracking parabolic dish concentrator is employed. The reactor is made up of an embedded proton exchange membrane (EC stack) within the reactor unit, a triple-junction III–V PV module, an aperture with a flux homogenizer, a shield, and a water pump for water recirculation over the PV. Figure 4a depicts a detailed illustration of the integrated photoelectrochemical (IPEC) reaction unit.

Because of its novelty and pilot-scale nature, this system includes a number of non-trivial design aspects. Specifically, it uses a two-pump architecture (global and PV recycle), allowing for independent regulation of the water flow rates for the EC and PV. This ensures that the CPV heat exchanger operates efficiently by transferring heat while keeping the EC’s stoichiometric water ratio and desired water temperature constant. Furthermore, in this pilot-scale arrangement, the size of the solar dish is not optimum for the reactor’s dimensions, necessitating the use of an additional water-cooled shield to control surplus focused sunlight. A concentrated photovoltaic unit or larger-area homogenizer could take the place of this shield. Figure 4b contains photos of the solar dish system that display the reactor unit out of direct sunlight as well as the entire system in operation. Despite being larger than required for the reactor, the 7-m-diameter parabolic dish was chosen for this experiment since a commercial dish with a similar size was readily available. The entire system was operated for more than 13 days, from the month of August 2020 to the months of February and March 2021. Main parameters that were measured are displayed in Fig. 4c. The system was used in a variety of weather conditions, with August’s average temperature of around 20 °C and late February’s average temperature of about 8 °C. The direct normal irradiance (DNI) statistics in Fig. 4c show that the weather conditions also changed during the operational periods. For instance, on August 19, 2020, the sky was clear with sporadic cumulus clouds, but on February 23, 2021, the upper atmosphere was covered in homogenous translucent clouds (such as cirrostratus).

With an EC current of IEC = 41.3 A, the instantaneous H_2_ generation rate is shown in Fig. 4b, which reaches a maximum of 0 9 Nm^3^ h^−1^ and averages 0.59 Nm^3^ h^−1^ (49.7 g h^−1^) over the course of operation. Utilizing the EC current, this rate was calculated and confirmed by gas chromatography under the assumption that proton exchange membrane (PEM) electrolysis has a high Faradaic efficiency. The total water flow rate was 4.92 L per minute, and the water flow rate for PV recycling was 10.3 L per minute. This means that the EC had a stoichiometric water ratio (λ) of 460 at 60 A. The system operated between 1 and 31 bar in the hydrogen storage tank, depending on the condition of storage, and at anodic side absolute pressures of around 3.5 bar and cathodic side absolute pressures of about 29 bar. The reactor’s average output temperature was between 60 and 70 °C, while the system’s usual water temperatures at the input and exit ranged from 14.4 to 45.1 °C.

Figure 4d shows the instantaneous input and output powers for the thirteen-day examination period. To get daily averaged performance metrics, these values can be combined throughout the day. Based on the reaction enthalpy (higher heating value of 286 kJ mol^−1^), the average system heat efficiency was 35.3% and the overall system fuel efficiency for the duration of the experiment was 6.6% ± 0.6%. By using the Gibbs free energy (237 kJ mol^−1^) as a benchmark, 5.5% ± 0.5% was found to be the system fuel efficiency. The external power that each auxiliary component needs is factored into the system efficiency, which is detailed under Methods. The fuel economy remained relatively constant throughout the winter, while the heat efficiency decreased. This was most likely caused by greater heat loss as a result of the dropping outside temperatures.

Over 3.2 kg of sunlight-generated hydrogen were produced over the entire experiment, and the highest rate of hydrogen generation reported in a 5-min period was 14.0 NL per minute (1.26 g min^−1^). According to the Methods, the system produced 10.6 kWth of thermal heat on average at an exit temperature of 45.1 °C. Over the course of the 13 days of operation, 679 kWhth of heat output was produced, with the peak output occurring during a 5-min interval at 14.9 kWth. The plant’s balance was made simpler and an auxiliary heater was eliminated thanks to the noteworthy reduction in auxiliary electrical demand caused by thermal integration.

As a result of the sacrifices made during the pilot-scale implementation, some components are not optimized, which lowers the overall efficiency below what is theoretically possible. Notably, the light shield absorbs too much concentrated light due to the huge dish. In the experiment, the diagnostic device’s efficiency is assessed by computing the ratio of fuel power to incident solar power on the reactor aperture (η_IPEC_ = *Q*_fuel_/*Q*_PV_). This evaluation is conducted using laboratory-scale thermally integrated PV–EC devices. An estimate of 27.5% was obtained by calculating the *Q*_solar_ to *Q*_PV_ ratio using Lambertian flux target calibration.

In summary, the diagnostic device exhibits an average efficiency of 24.4% ± 2.8% throughout the testing campaign, reaching 27.2% on its most successful day. These values, based on Gibbs free energy, become 20.3% ± 2.3% and 22.6%, respectively. When it comes to solar-to-fuel efficiency and energy output, this system performs well in comparison with earlier studies, particularly those that used thermal integration of photovoltaic and proton exchange membrane electrolyzer (PEM-EC) components. Notably, this work was accomplished in real-world solar settings instead of laboratory-simulated surroundings, and it also shows a significant improvement in solar hydrogen generation power when compared to earlier results. Advancements in these developments can be attributed to the utilization of cutting-edge triple-junction photovoltaic materials and enhanced efficiency in the coupling of solar and electrochemical processes. The study’s achieved efficiencies are among the highest in the field of solar hydrogen production and co-generation systems.

### PEC/PEC Tandem Cell Water Splitting

**Fig. 5:**
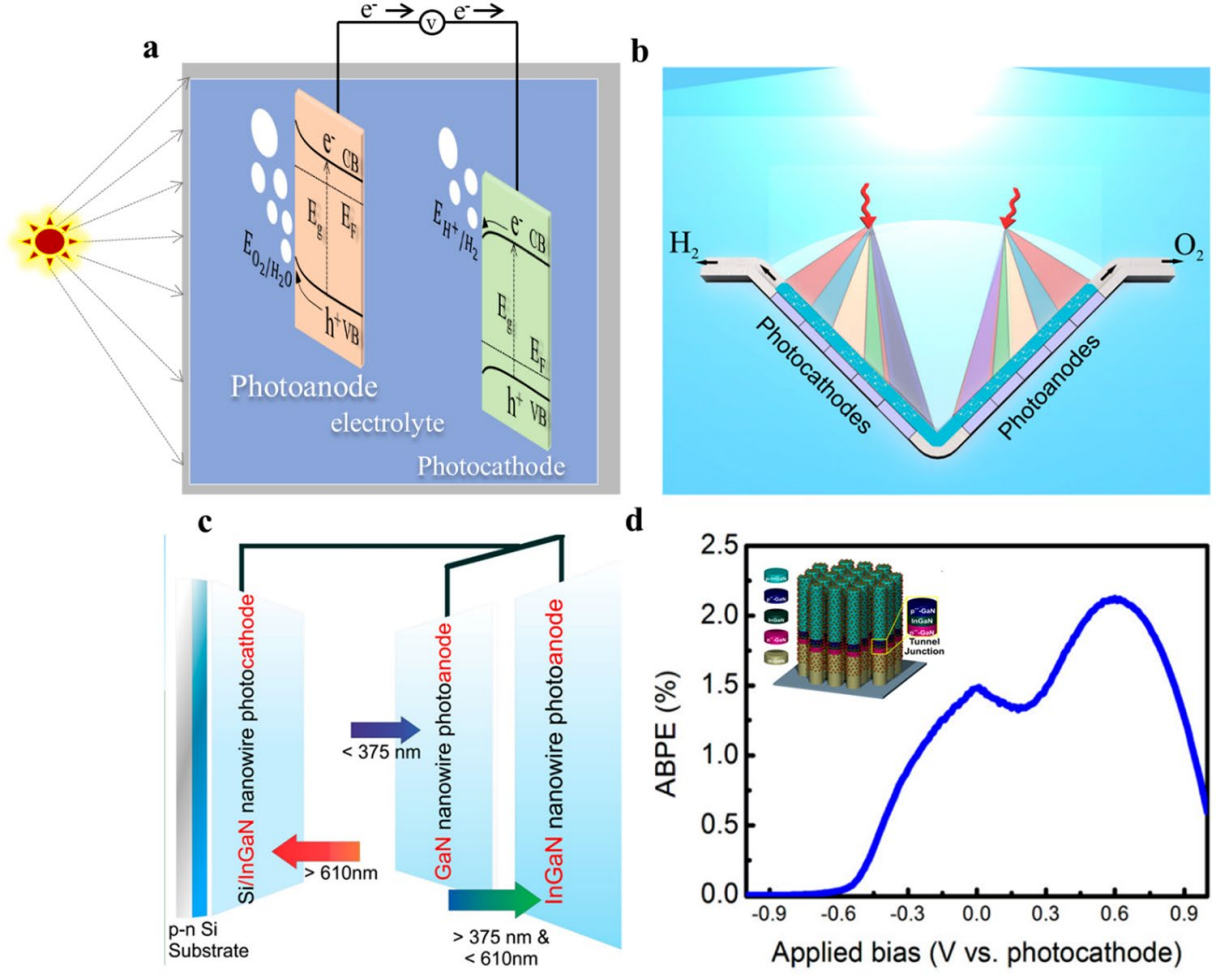
Tandem photoelectrochemical cell configuration. **a** Illustration outlining the configuration of the photoelectrochemical tandem cell. **b** Conceptual representation of a dual-photoelectrode system with parallel illumination. The incident solar spectrum is spatially and spectrally split on the photoanode and photocathode. Each electrode, forming the photoanode or photocathode, is parallel-connected and illuminated with a specific portion of the solar spectrum. **c** Schematic representation of a parallel-connected photoanode composed of GaN and InGaN nanowires, along with a Si/InGaN photocathode. Incident sunlight is spectrally and spatially distributed among these photoelectrodes. **d** Power conversion efficiency of the dual-photoelectrode device (as depicted in **b**) plotted against applied bias under AM1.5G 1 sun illumination. The maximum power conversion efficiency is estimated to be 2% at 0.6 V vs. photocathode. Reproduced with permission from Ref. [148]. Copyright 2015 Springer Nano Letters

Using a tandem cell to create a PEC/PEC tandem cell by linking a photocathode and a photoanode in succession is an efficient method for overall water splitting. Sunlight passes through the n-type semiconductor in this setup, followed by the p-type semiconductor. Oxidation of water on the n-type semiconductor surface yields oxygen, whereas reduction processes on the p-type semiconductor surface yield hydrogen [147]. In particular, the top electrode in this configuration, the photoanode, absorbs photons in the shorter wavelengths of the solar spectrum, while the bottom electrode, the photocathode, transmits and collects the remaining longer-wavelength photons. This configuration maximizes the use of solar photons by enabling numerous stages of absorption. From a thermodynamic point of view, it is more advantageous to employ two semiconductors with smaller band gaps than one photoelectrode as each photoelectrode only needs to supply a portion of the total potential for water splitting [147]. Each photoelectrode’s band gap should span the O_2_/H_2_O or the H^+^/H_2_ standard electrode potential, as shown in Fig. 5a, with the photoanode’s conduction band being more negatively oriented than the photocathode’s valence band. More photons may be absorbed by semiconductor materials with smaller band gaps, which increases STH efficiency. Dual-photoelectrode devices based on III-nitride nanowires (NWs) for total solar water splitting have been reported by researchers AlOtaibi et al. [148]. A monolithically integrated p-InGaN NWs/tunnel junction/Si photocathode that joins parallel GaN NWs and InGaN NWs photoanodes makes up the tandem structure, as shown in Fig. 5b. The GaN photoanode, InGaN photoanode, and photocathode are all individually exposed to wavelengths of less than 375, 375–610, and greater than 610 nm. This configuration efficiently partitions the sun spectrum to meet the photon absorption needs of single-bandgap photoelectrodes. Figure 5c shows the general idea of this dual-photoelectrode system with parallel illumination.

The dual-photoelectrode system, which consisted of an InGaN nanowire photocathode and a GaN nanowire photoanode, demonstrated an approximately 20-fold increase in power conversion efficiency under parallel illumination (400–600 nm) and an open-circuit potential of 1.3 V when compared to individual photoelectrodes. Anode-based photoelectrochemical efficiency (ABPE) of 2% at 0.6 V vs. the photocathode was also attained by a monolithically integrated single-junction Si/InGaN nanowire photocathode and parallel-connected metal-nitride nanowire photoanodes in the dual-photoelectrode configuration, as shown in Fig. 5d. High open-circuit potential is provided by the big bandgap photoelectrode, and photocurrent matching is easily accomplished by separating the solar spectrum in proportion to band gap engineering. Furthermore, by joining many photoanodes (also known as photocathodes) in parallel, the photovoltage and photocurrent can both be maximized. When combined with the parallel illumination scheme, metal-nitride nanowire photoelectrodes have the extraordinary potential to achieve high efficiency, unassisted solar-to-hydrogen conversion. This is demonstrated by the significantly improved power conversion efficiency, which is unprecedented for a dual-photo-electrode device.

### PEC/EC Switchable Water Splitting

**Fig. 6:**
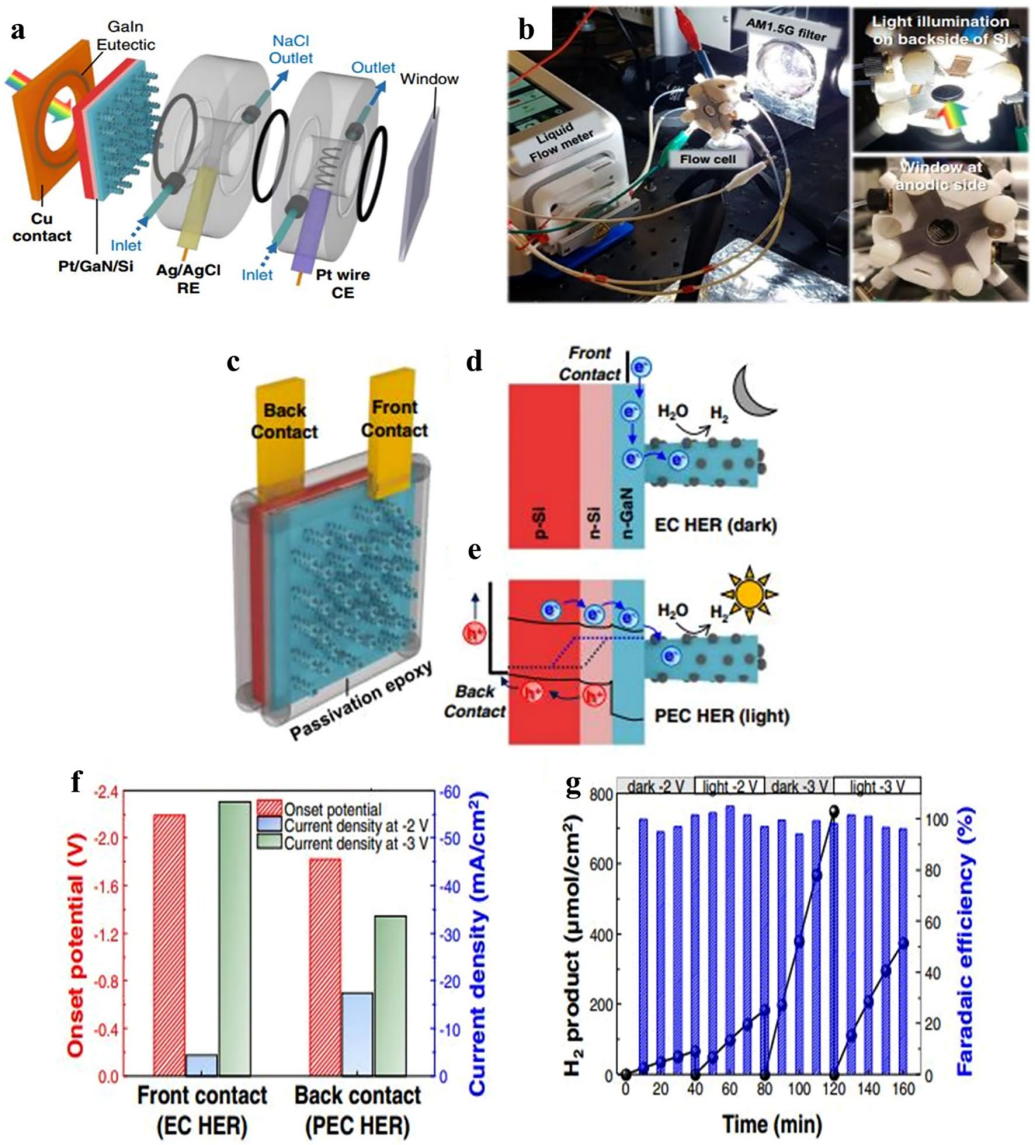
Switchable electrode for PEC/EC. **a** Focused solar light induced Phot electrochemical water splitting schematic picture. **b** Snapshots of the PEC water splitting liquid flow cell. **c** Switchable dual contact electrode schematic. For the Ohmic connection, the front contact was positioned on the front side of GaN nanowires (NWs), and the rear contact was positioned on the back side of the n + -p Si wafer, with the GaIn eutectic sandwiched between the Cu contacts and the substrate. Illustrations of the operating principles of **d** electrochemical HER in the dark (front contact) and **e** photoelectrochemical HER in the light (rear contact), **f** the onset potential and current density (at − 2 & − 3 V) vs front and back contact HER graph. The potential at − 10 mA cm^−2^ was identified as the onset potential. **g** Measurements of the amount of H_2_ generated and faradaic efficiency in the presence of light (back contact) and darkness (front contact). Reproduced with permission from Ref. [ 149]. Copyright 2015 Springer Nature Communications

Among many techniques, water electrolysis is a possible path toward low-temperature, high-purity H_2_ production. PEC HER photocurrent can be increased by using concentrated solar light, which increases photogenerated electrons. However, high H_2_ evolution under concentrated solar light may impede reaction kinetics because of restrictions on mass transfer and gaseous bubbles shielding the active site. These problems are solved by a flow cell, which makes it possible for reagent to be delivered quickly and for gas bubbles to physically separate from the electrode surface [149]. In order to demonstrate high photocurrent density, we built a liquid flow cell reactor for concentrated solar light studies in Fig. 6a. Figure 6b shows snapshots of the PEC water splitting liquid flow cell.

The PEC HER transpired on the front side of the reactor, and light was incident on the backside of an Si wafer. Prepared GaN nanowires (NWs) with a high density of Pt nanocrystals (NCs) were highly conductive, n-type, and degenerately doped. This made it possible for the GaN NWs/Si wafer’s front side to have electrical contact and serve as an electrochemical HER cathode. Consequently, it was demonstrated that Pt/GaN/Si could operate in both light and dark conditions by simply adjusting the placements of the contacts (Fig. 6c). During electrochemical HER in the dark, H_2_ developed as a result of the electrical bias pumping electrons into GaN NWs (Fig. 6d). In contrast, the Si substrate in PEC HER, which has a low bandgap of approximately 1.1 eV, generated electron–hole pairs in response to solar radiation (Fig. 6e). Photogenerated electrons in the conduction band moved toward n-type GaN NWs and Pt NCs for HER, while holes moved to the back contact and counter electrode to participate in the anodic process.

The performance of the switchable electrode is evaluated in a 0.5 M NaCl solution. No photocurrent is observed when the rear contact, or PEC mode, is used. Under one solar light (1 sun) irradiation, the overpotential (η_10_) is 0.11 V vs. the reversible hydrogen electrode (RHE) in a three-electrode setup and − 1.83 V in a two-electrode setup. In comparison with the electrochemical (EC) process, the Si p–n junction’s own potential favorably shifts the onset potential about roughly 0.4 V. This results in a low operating voltage (− 2 V) and a relatively high current density (− 17.5 mA cm^−2^) (Fig. 6f). The quantity of minority carrier propagation in the opposite direction and photogenerated charge carriers, which saturate at about 35 mA cm^−2^, however, limits the photocurrent density. In contrast, a higher amount of H_2_ (− 58 mA cm^−2^) at − 3 V is obtained during EC HER with the front contact in the dark. Majority carriers can be employed for HER due to the high-quality, degenerately doped n-type GaN NWs, which prevent Fermi level pinning and provide efficient electron transport to Pt at the non-polar sidewalls. This is supported by the fact that the Pt/GaN/Si assembly exhibits a higher saturation photocurrent density than Pt/Si.

The H_2_ production and faradaic efficiency of Pt/GaN/Si at − 2 and − 3 V in both bright and dark conditions are shown in this Fig. 6f. The productivity and faradaic efficiency of Pt/GaN/Si under dark and light conditions are measured and shown in Fig. 6g. The production rate in light and dark conditions at − 2 V was 276 and 100 μmol cm^−2^ h^−1^, respectively, suggesting that PEC HER was preferred over EC HER under light conditions. In contrast, the rate of electrochemical hydrogen evolution reaction (EC HER) reached approximately double that of photoelectrochemical hydrogen evolution reaction (PEC HER), measuring 1125 and 562 μmol cm^−2^ h^−1^, respectively, particularly at a more negative potential of − 3 V. Importantly, the faradaic efficiency remained nearly 100% in all scenarios. Consequently, the Pt/GaN/Si assembly exhibited the capability to consistently generate clean hydrogen by functioning as an electrocatalyst during nighttime and as a photocathode during daytime.

A synergistic binary system exhibiting highly effective catalytic hydrogen evolution over a broad pH range was found for Pt/GaN on Si electrodes. Excellent overpotentials (η_10_) of 0.15, 0.16, and 0.39 V vs. RHE were shown by Pt/GaN/Si, in that order. It also showed excellent anode-based photoelectrochemical efficiency (ABPE) in phosphate-buffered seawater (pH = 7.4), 0.5 M NaCl (pH = 9.1), and seawater (pH = 8.2). Under intense solar light (nine suns), a 2-electrode configuration photocathode ran continuously for more than 120 h, reaching an all-time highest photocurrent density of about − 169 mA cm^−2^. These stability and efficiency values for PEC HER in seawater are among the greatest yet recorded. Pt/GaN/Si most notably showed off its exceptional capacity to operate in both the dark (EC) and the light (PEC). These results demonstrate that the Pt/GaN/Si assembly outperforms traditional photoelectrolytic systems in promoting catalytic processes and can function as an energy-saving electrode.

### Catalyst-Free InGaN QPs-NWs PEC Water Splitting

**Fig. 7:**
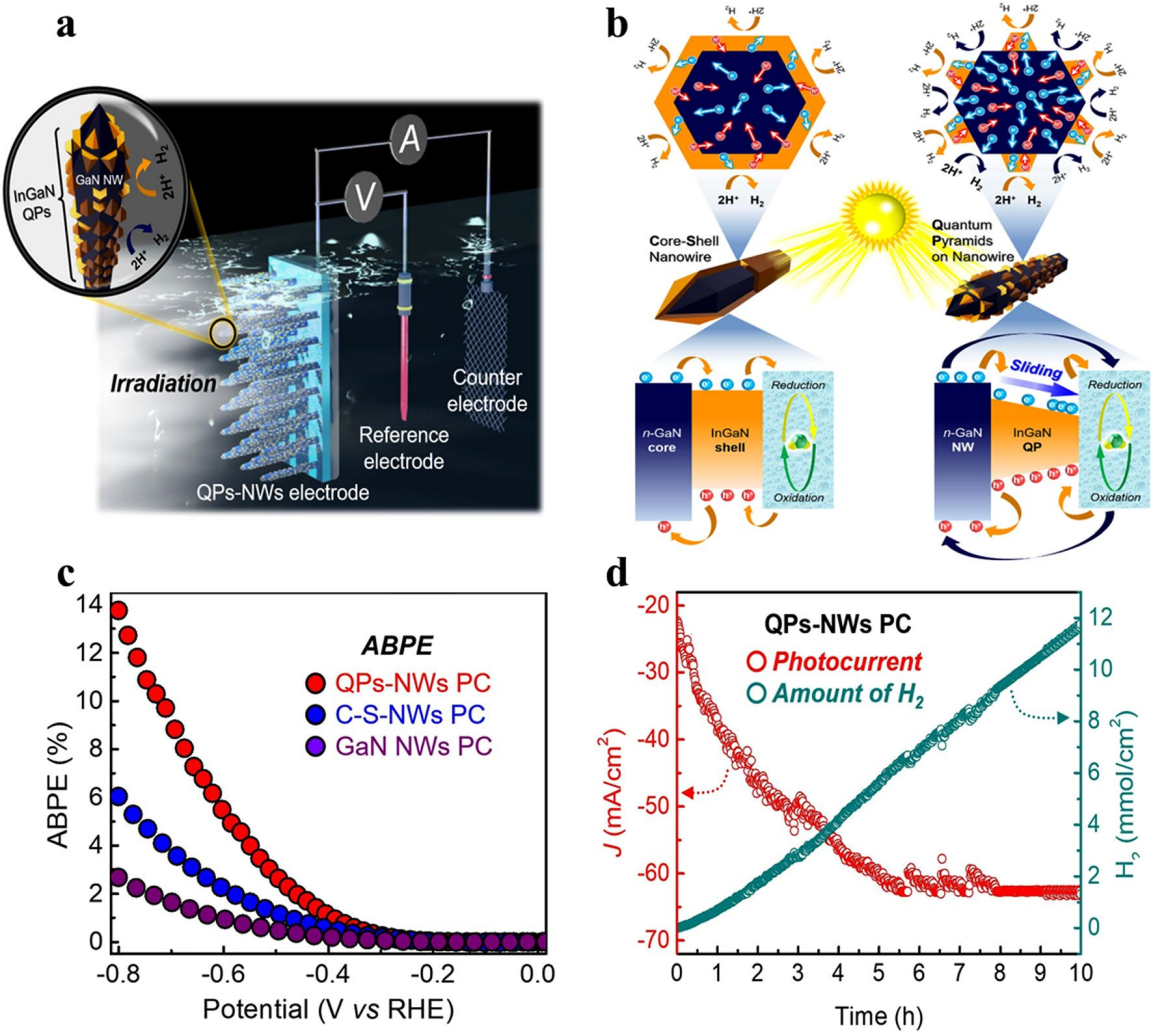
Photoelectrochemical Water Splitting Cell with InGaN/GaN quantum dots-nanowires photocathode. **a** Diagram illustrating the photoelectrochemical water splitting (PEC-WS) cell with an InGaN/GaN quantum dots-nanowires (QPs-NWs) photocathode. **b** Schematic diagrams depicting the top view of the oxidation–reduction process of InGaN/GaN core–shell nanowires (C-S-NW) and InGaN/GaN QPs-NW, explaining their respective photogenerated electron–hole pair mechanisms. Energy diagrams of InGaN/GaN C-S-NW and InGaN/GaN QPs-NW highlight their unique charge carrier transport mechanisms. **c** Anodically biased photoelectrochemical (ABPE) data derived from current–voltage (*J–V*) curves of GaN nanowires (NWs), C-S-NWs, and QPs-NWs. **d** Hydrogen generation performance for QPs-NWs photocathode at − 0.5 V versus reversible hydrogen electrode (RHE) under illumination in 0.5 M H_2_SO_4_ for a duration of 10 h. Reproduced with permission from Ref. [152]. Copyright 2015 Springer Chemical Engineering

The integration of InGaN/GaN quantum pyramids on nanowires (QPs-NWs) presents a viable remedy for the decline in photocathode (PC) performance. These new InGaN QP structures on non-polar GaN nanowires provide a unique opportunity to control their energy band gap (*E*_g_) between around 2 and 1.36 eV because of the quantum sliding interface recombination effect [150, 151]. Due to this amazing property, QPs-NWs PCs can perform photoelectrochemical water splitting (PEC-WS) without the assistance of outside catalysts. The QPs-NWs devices with a double-band photoelectrode shape are functional and have an exceptionally high incident photon-to-current conversion efficiency. As shown in Fig. 7a, the photocathodes, InGaN/GaN C-S-NWs and QPs-NWs, which have a high indium content of 40–65% and excellent quality, were assessed for possible application in PEC-WS (photoelectrochemical water splitting) technology. Figure 7b shows a schematic representation of the PEC-WS process and shows the top view of the redox reaction at the photocathode surface together with the flow of generated electron–hole carriers. Charge transfer takes place during this procedure when the PEC cell is submerged in an electrolyte. At the interfaces of the electrolyte/InGaN/GaN, an initial upward band bending occurs to preserve an equilibrium state free from biasing. The energy band diagram makes it very evident that only the InGaN shell participates in the charge transfer process in QPs-NWs photocathodes, but both InGaN QPs and GaN NWs participate in C-S-NWs photocathodes. During the PEC-WS redox process in the InGaN/GaN C-S-NW PC, photogenerated holes migrate from the InGaN shell to the GaN core and photogenerated electrons migrate from the GaN core to the InGaN shell. High surface-to-volume ratio InGaN QPs with 3D pyramidal morphologies are advantageous to the QPs-NWs structure in terms of charge carrier generation and effective photon absorption. They exhibit a broad variety of absorption spectra as well. Furthermore, the energy band sliding mechanism, which raises electron mobility, improves the water splitting (WS) performance in the reduction reaction.

After examining the current–voltage (*J–V*) characteristics of GaN NWs, C-S-NWs, and QPs-NWs photocathodes within the voltage range of − 0.8 to 0 V relative to the reversible hydrogen electrode (RHE), Fig. 7c shows the incident photon-to-current conversion efficiency (ABPE) against potential curve. With an ABPE of nearly 13.75%, the QPs-NWs photocathode was the highest performing one, while the C-S-NWs photocathode yielded the highest value of 6.03%. In contrast to RHE, GaN NWs displayed a notably reduced ABPE of 2.67% at − 0.8 V. These findings offer empirical proof of the powerful crystallinity and efficient charge carrier generation capabilities of the manufactured PCs. However, due to photocurrent saturation, achieving further increases in ABPE beyond an applied bias of − 0.8 V is unlikely. This saturation is attributed to factors such as limited mass diffusion of protons at the interface between the liquid and the semiconductor, as well as bubbling resistance. To tackle the main issue with PEC-WS technology in practical applications, which is the noticeable electrode deterioration, Fig. 7d shows that after 6 h, the QPs-NWs PC’s current density stabilized at around 61.81 mA cm^−2^. The QPs-NWs PC was predicted to produce 11.5 mmol cm^−2^ of H_2_ in ten hours. These findings imply that the PC’s architecture provides a useful and effective way to incorporate high-performance PEC-WS technology into practical applications.

### PEC/PV Cell Water Splitting

**Fig. 8:**
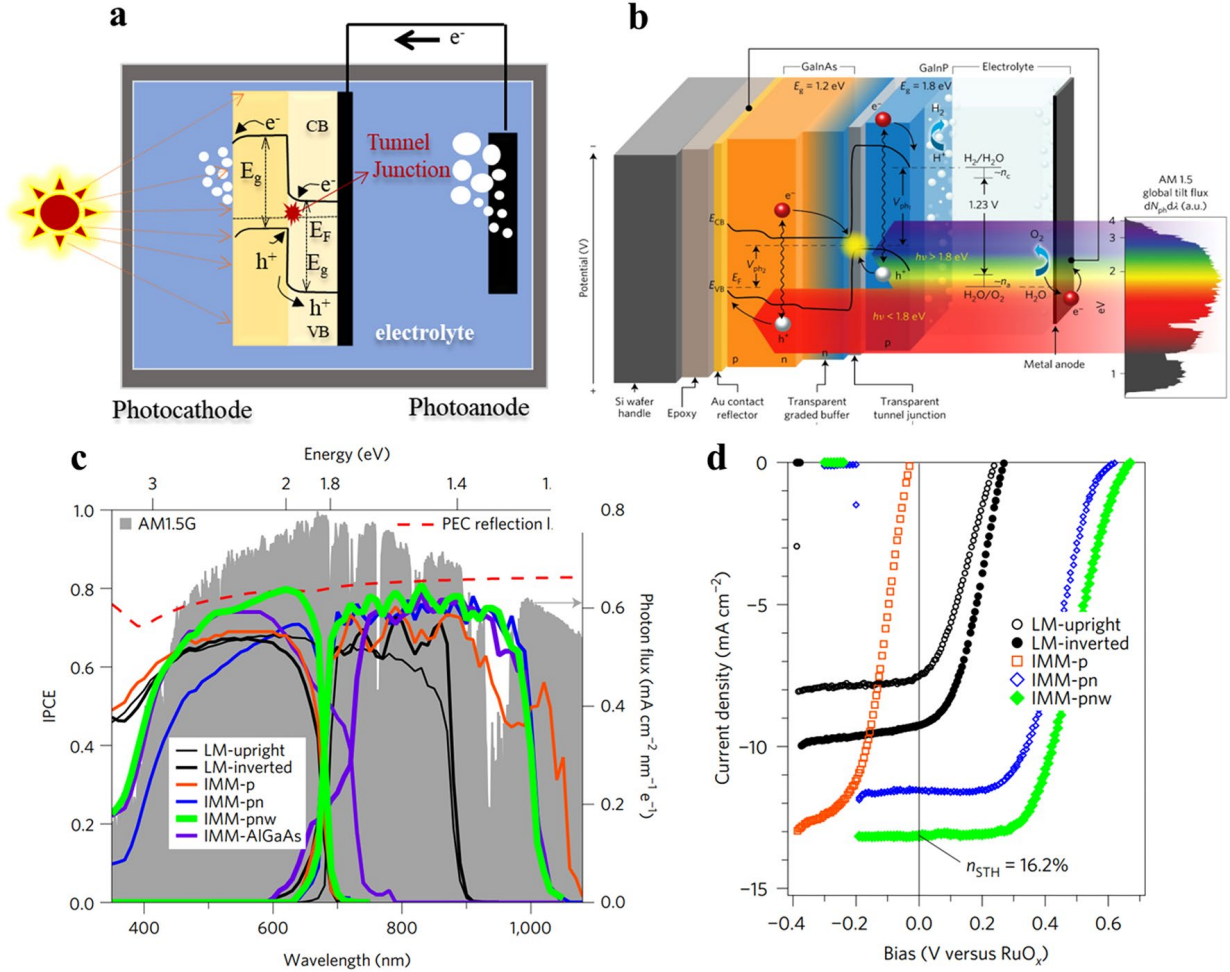
Presents a detailed depiction of a PEC/PV tandem cell setup. **a** Schematic illustrates the configuration, while **b** provides an overview of an IMM photocathode designed for water splitting. The IPCE data in **c** is superimposed on the AM1.5G reference spectrum and the PEC reflection limit. Additionally, **d** showcases *J–V* measurements conducted on-sun under collimated, direct-only solar illumination, with results plotted and translated to AM1.5G reference conditions. Reproduced with permission from Ref. [153]. Copyright 2015 Springer Nature Energy

A different and workable method to accomplish unassisted total water splitting is to build a PEC/PV tandem cell by fusing photovoltaic cells with photoelectrodes. With an integrated PV device, this tandem cell functions as a voltage-biased PEC device [154]. The PEC component of the photoelectrodes in this design involves the semiconductor material’s minority carriers participating in the water redox reaction at the semiconductor-electrolyte junction. The majority carriers (electron) produced in the photoanode recombine in the PV cell through the external circuit; n-type semiconductor materials are one example of this. At the metal counter electrode attached to the PV cell, hydrogen is produced, as seen in Fig. 8a. When minority carriers are unable to provide enough power, solar cells become essential to the operation. The bias produced by the solar cell removes the requirement for matching energy levels and increases flexibility in the selection of PV cells and photoelectrode materials. The system relies solely on light as the energy input, aligning with the PEC/PEA architecture. Other examples involve immersed gadgets in addition to the previously described external device. In 1998, Turner created a noteworthy design that combined a GaAs p–n junction with a GaInP_2_ photocathode, resulting in a 12.4% efficiency in producing hydrogen. Nevertheless, this system had drawbacks, such as the GaInP_2_ photocathode’s quick corrosion when it came into contact with the aqueous electrolyte because of its pricy and hazardous components, which rendered it unsuitable for large-scale applications.

In order to achieve a STH efficiency of more than 16%, Deutsch’s group in 2017 produced another submerged arrangement that used a buried p–n junction with GaInP/GaInAs tandem absorbers [155]. Higher energy photons in this configuration are absorbed by GaInP, while lower energy photons pass through to the GaInAs junction at the bottom. GaInP conduction band electrons contribute to the creation of H_2_, whereas GaInAs valence band holes produce O_2_ when they come into contact with a metal counter electrode. Compared to previous solid-state solar cells, this device, known as an inverted metamorphic multi-junction (IMM), reduces the amount of external wiring and may therefore save expenses. The incident photon-to-current efficiency (IPCE) of each of the six devices is shown in Fig. 8c, superimposed on the AM1.5G reference spectrum. Higher STH efficiency can be achieved using the flexible platform that the IMM device offers for achieving tandem band gap combinations. Interestingly, GaAs is found to be the limiting junction; to increase the spectral sensitivity of the bottom junction, future enhancements could replace GaAs with narrower bandgap GaInAs alloy(s). The ideal bandgap, enhanced spectral response, stability, and anti-reflection can be achieved by integrating absorbers that have been independently created with flexibility thanks to the IMM design. Future research might concentrate on creating stable, perfect 1.7 eV top bandgap arsenide-based III–Vs (such GaInAsP [155]) without aluminum, or it could solve reflection losses and create a 1.05−eV bandgap bottom junction to improve tandem device η_STH_ chances toward 27%.

## Recent Advances in Solar CO_2_ Reduction

### Flue Gas CO_2_ Reduction

The PEC CO_2_ RR (Reduction reaction) uses clean sources of solar energy, water, and CO_2_ at a semiconductor/water interface to create chemical fuels like formic acid (HCOOH). The initial stage in the PEC CO_2_ RR is the production of electron–hole pairs in semiconductors by the absorption of incoming photons [156]. So far, most research efforts have been focused toward the creation of photocathodes using pure CO_2_ as a feedstock. However, for practical applications, it is crucial to consider its compatibility with flue gas CO_2_. Flue-gas CO_2_ often contains various impurities like H_2_, NOx, CO, and H_2_S compounds [157–159]. Consequently, there is considerable interest in converting impurity-tainted flue-gas CO_2_ into chemicals that are useful without requiring costly purification processes.

**Fig. 9:**
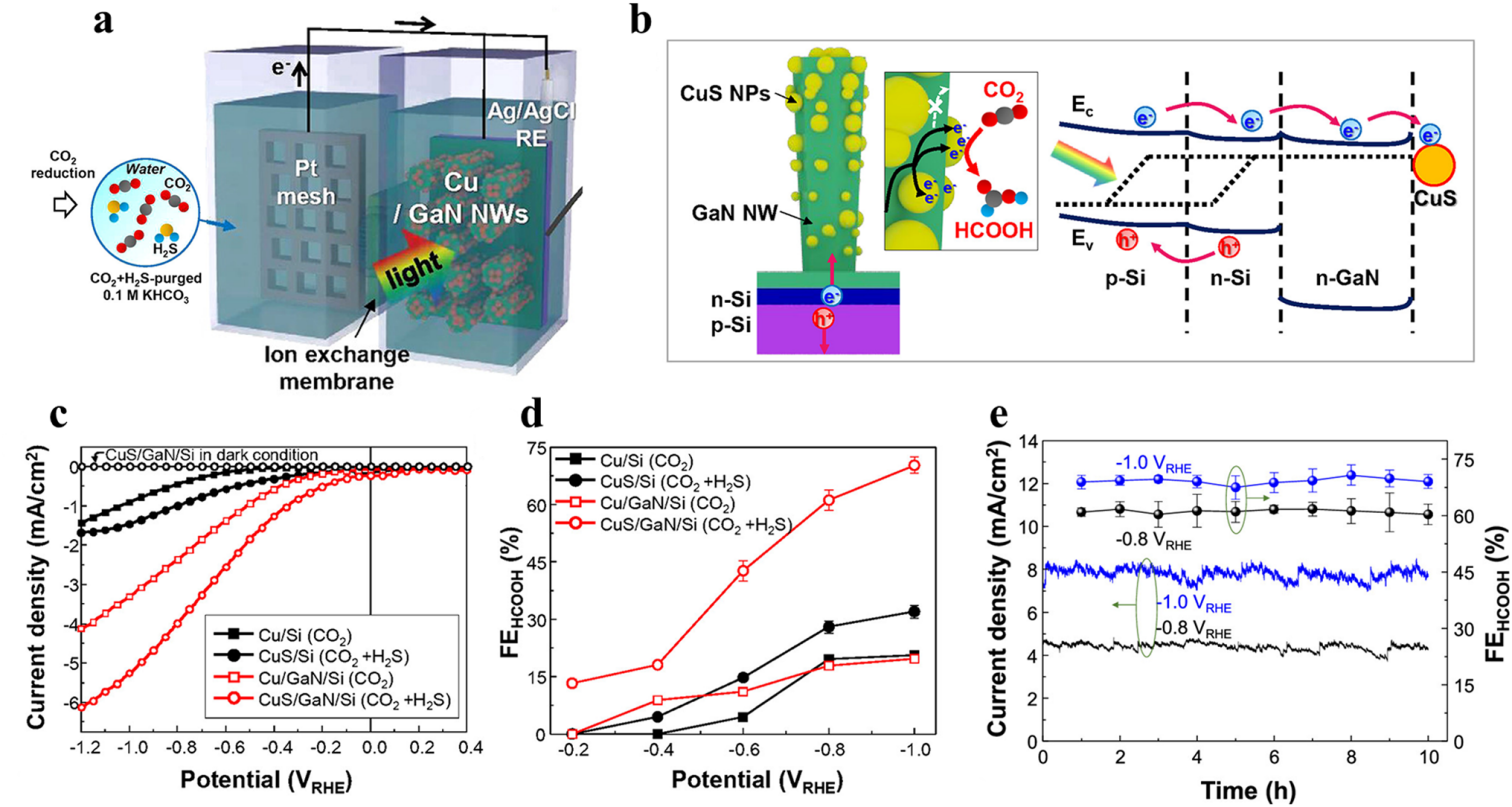
Provides a schematic representation of **a** PEC CO_2_ reduction and **b** the energy band diagram of a CuS/GaN/Si photocathode. The illustration in **a** outlines the process of photoelectrochemical carbon dioxide reduction, while **b** presents the energy band diagram specific to the CuS/GaN/Si photocathode. The electrochemical performance is further demonstrated through **c** linear sweep voltammetry (LSV) curves and **d** Faradaic Efficiency (FE) measurements of formic acid (HCOOH) for photocathodes evaluated in electrolytes purged with either CO_2_ or CO_2_ + H_2_S. Additionally, **e** showcases the stability of the CuS/GaN/Si photocathode at − 0.8 and − 0.1 V vs. the reversible hydrogen electrode (RHE). Reproduced with permission from Ref. [160]. Copyright 2021 Springer, The American Chemical Society

A novel discovery offers various architecture for the efficient and dependable production of formic acid (HCOOH) from CO_2_ mixtures containing H_2_S impurities: a photocathode with a CuS cocatalyst atop GaN nanowires/n + –p Si. Cu nanoparticles that were initially formed on GaN nanowires transform into CuS nanoparticles when H_2_S impurities dissolve in the electrolyte during the CO_2_ reduction reaction (Fig. 9a). When the Si substrate is photoexcited by solar radiation, the method generates electron–hole pairs for it, with a small bandgap of about 1.1 eV (Fig. 9b). GaN NWs show very poor light absorption due to their large bandgap (∼3.4 eV); yet, they decrease Fresnel reflection, increasing the light absorption of the flat Si substrate. Their unique design helps to equalize the air’s and the Si substrate’s refractive indices. Furthermore, gallium nitride nanowires (GaN NWs) serve as efficient geometric modifiers for incorporating copper sulfide nanoparticles (CuS NPs) into cocatalysts, thereby amplifying and improving the catalytic process. The electrochemical surface area of CuS NPs on GaN NWs is approximately 16.8 times more than that of a planar Si wafer. This architecture allows for the spatial decoupling of the catalytic activity and light harvesting, allowing for the optimization of both optical and catalytic properties for maximum performance. As seen by the electrode’s energy diagram (Fig. 9b), electron transport is also improved without encountering an energy barrier between GaN NWs and the Si substrate because both are strongly n-type doped and have roughly aligned conduction band edges.

In electrolytes purged with either CO_2_ or CO_2_ + H_2_S, a photoelectrochemical CO_2_ reduction process was carried out to assess the effect of Cu and CuS cocatalysts on photocathodes. In comparison with Cu/Si and CuS/Si, GaN/Si cocatalysts paired with Cu and CuS cocatalysts produced on n + –p Si showed a considerable boost in onset potential and photocurrent density (Fig. 9c). When Cu cocatalysts were converted into CuS on an Si photocathode at − 1.0 V RHE, the selectivity for HCOOH rose from roughly 21% to roughly 32% (Fig. 9d). Interestingly, under the same conditions, the Faradaic efficiency (FE) of HCOOH increased significantly from approximately 20% to over 70% when Cu was transformed into CuS nanoparticles on GaN nanowires. This suggests that the photoelectrochemical CO_2_ reduction reaction can be enhanced rather than degraded by the addition of H_2_S impurity to CO_2_ gas.

CuS/GaN/Si consistently demonstrated photocurrent densities of around 4.5 and 7.8 mA cm^−2^ at − 0.8 and − 1.0 V vs. RHE, respectively, yielding significant Faradaic efficiencies (FEs) of approximately 60% and 70% (Fig. 9e). The extraordinarily stable, unique, N-rich GaN surface is explained by its resistance to sulfurization and oxidation by flue-gas CO_2_ contaminants.

### High-Purity CO_2_ Gas CO_2_ Reduction

In comparison with energy-intensive and complex electrocatalysis, thermocatalysis and biocatalysis, photocatalysis has emerged as a promising technology for carbon fixation. Its simplicity of design, affordability, and environmental consciousness are what make it appealing. It is very interesting to achieve the photocatalytic synthesis of C_2+_ compounds without the need for sacrificial agents, notwithstanding the difficulties involved. A range of photocatalytic devices have been investigated for the reduction of CO_2_; copper and its compounds are known to be effective in the synthesis of C_2+_ compounds [160–162]. Through surface reconstruction, oxidation-state tuning, and defect engineering, a variety of C_2+_ compounds can now be produced on copper-based catalysts. In contrast to electrocatalysis, photocatalysis provides a more approachable method of producing C_2+_ molecules from CO_2_ and H_2_O without the need for applied potentials.

**Fig. 10:**
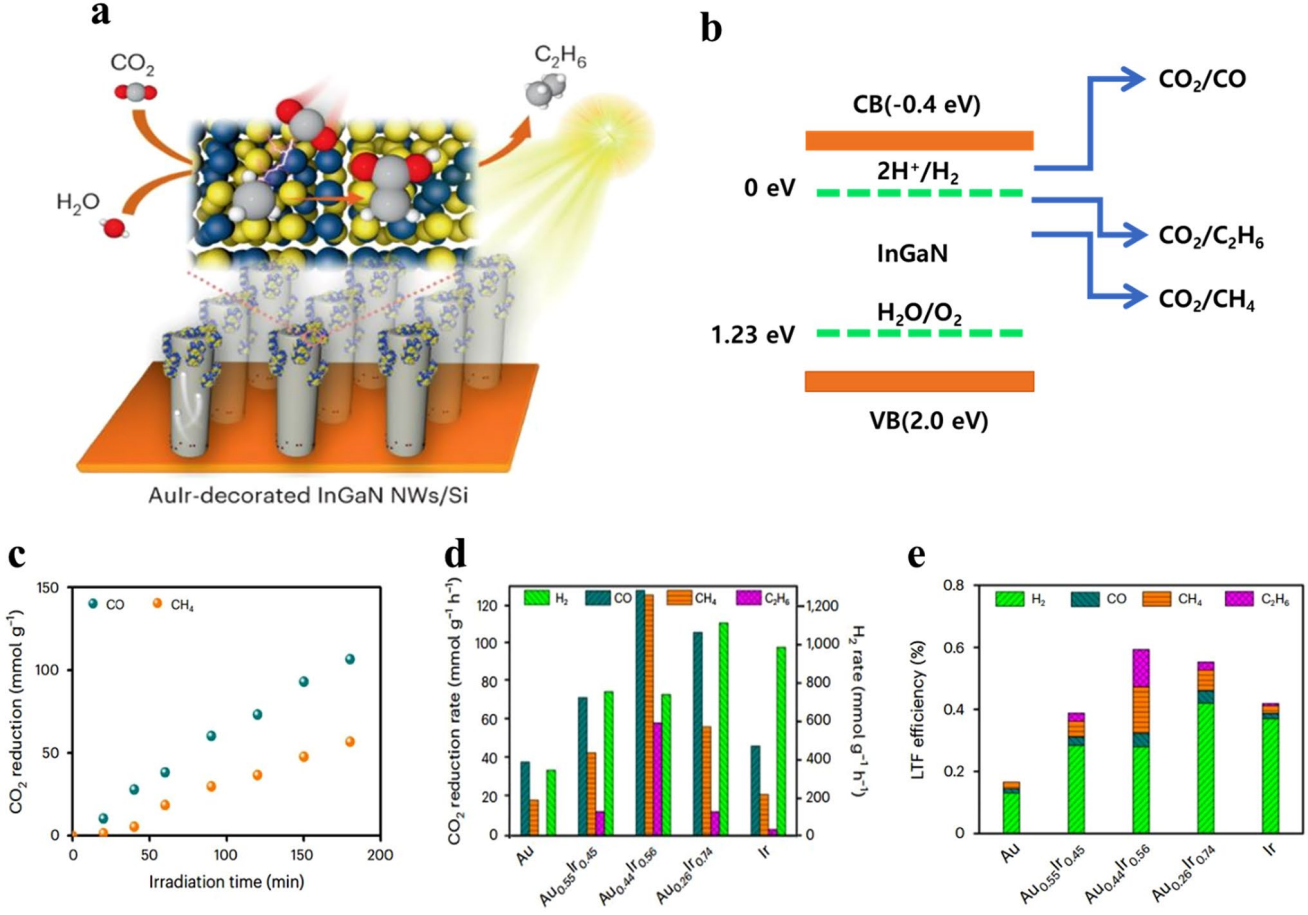
**a** Schematic of light-driven C–C coupling from CO_2_ and H_2_O over Au_0.44_Ir_0.56_@ InGaN NWs/Si. The illustration outlines the process of carbon–carbon coupling under light irradiation using Au_0.44_Ir_0.56_-decorated InGaN nanowires on silicon. **b** Includes a SEM (scanning electron microscopy) image and energy band structure of InGaN nanowires on silicon, where CB represents the conduction band and VB represents the valence band. **c** Displays the time course of CO and CH_4_ evolution from the CO_2_ reduction reaction over Au_2_@ InGaN NWs/Si. Further details include the evolution rate **d** and LTF (long-term stability) efficiencies **e** of various products obtained from photocatalytic CO_2_ reduction over AuIr@InGaN NWs/Si with varying Au/Ir ratios. Reproduced with permission from Ref. [165]. Copyright 2021 The Nature Catalysis

To achieve carbon neutrality, an intelligent artificial photosynthesis integrated device that uses carbon dioxide, water, and sunshine to produce C^2+^ molecules is required. An essential precondition for this process is an effective catalyst to overcome the C–C coupling bottleneck [127, 128]. Feedstock tests, theoretical computations, and operando spectroscopic observations were used to get these important conclusions. It has been discovered that gold and iridium can catalyze the reduction of CO_2_ and enable C–C coupling by introducing CO_2_ into –CH_3_ groups [163]. The production of C_2_H_6_ from CO_2_ and H_2_O is catalyzed by the combination of Aulr and InGaN nanowires on silicon. According to Fig. 10a, iridium may act as a mediator between gold and iridium to facilitate C–C coupling through the insertion of CO_2_ into –CH_3_ [164, 166], thereby promoting the synthesis of C_2_H_6_ from CO_2_ and H_2_O over AuIr@InGa NWs/Si.

The energy band positions of InGaN nanowires are in good agreement with the required redox potentials for CO_2_ reduction with water, as shown in Fig. 10b. This favorable alignment enables the thermodynamic feasibility of producing C^2+^ compounds without the use of sacrificial agents or applied bias. The studies were conducted in a specifically designed glass container filled with an aqueous CO_2_ solution and lit with focused light at 3.5 W cm^−2^ without the use of sacrificial agents or bias. Without catalysts, InGaN nanowires do not produce carbon products and have very little hydrogen evolution activity. However, at varying quantities of gold supplied, InGaN nanowires on silicon exhibit significant CO_2_ reduction activity. CO is the main carbon product that gold predominantly creates, along with other C_1_ compounds. The characteristics of the gold nanoparticles determine their activity and selectivity (Au NPs). With gold loading, the rate of CO production rises and peaks at an average Au NP size of 6.0 nm, with a selectivity of 9.6%. At this point, CO_2_ reduction is less preferred than the hydrogen evolution process (HER), with H_2_ exhibiting strong selectivity (85.7%). Higher gold loadings result in worse CO performance, most likely because of lower catalytic activity as the Au NP size increases up to 10.4 nm.

The study investigated the temporal evolution of CO_2_ reduction products using optimized AuIr@InGaN NWs/Si to gain a comprehensive understanding of the reaction. In Fig. 10c, CO emerged as the primary product, accompanied by CH_4_ as a by-product. Pure gold showed no detection of C^2+^ compounds, confirming its incapacity for C–C coupling toward C^2+^ compounds. Notably, AuIr@InGaN NWs/Si outperformed Au NPs@InGaN NWs (Fig. 10d), with significantly improved overall activity upon the addition of iridium. AuIr@InGaN NWs demonstrated C_2_H_6_ synthesis activity, achieving a C_2_H_6_ evolution rate of 58.8 mmol g^−1^ h^−1^ with a selectivity of 5.6%. The total hydrocarbon selectivity, including CH4 and C2H6, reached 17.6%. Additionally, the formation of syngas (CO and H_2_) as a useful by-product exhibited appreciable activity and high selectivity (863.4 mmol g^−1^ h^−1^, 82.4%). Considering all photocatalysis products, Au_0.44_Ir_0.56_@InGaN NWs/Si achieved an LTF efficiency of 0.59%, 3.5 times higher than Au NPs@InGaN NWs/Si (0.17%). Ir@InGaN NWs also showed C_2_H_6_ formation activity, though with lower activity (3.3 mmol g^−1^ h^−1^) and selectivity (0.31%) than AuIr@InGaN NWs (Fig. 10e). The findings underscore the critical role of iridium in C–C bond formation, highlighting the indispensability of both AuIr and InGaN NWs. Under low light intensity, the reaction’s efficiency was limited, likely due to an insufficient number of photogenerated electron–hole pairs. However, under high light intensity (> 1.5 W cm^−2^), CO_2_ reduction efficiency sharply increased, with InGaN NWs photoexcited to generate abundant electrons and holes under concentrated light (3.5 W cm^−2^). The reaction proceeded through thermally assisted photocatalysis, with photogenerated charge carriers playing a pivotal role in the superior performance. In the absence of CO_2_, photoreduction by Au_0.44_Ir_0.56_@InGaN NWs/Si under vacuum produced only hydrogen, confirming that C^2+^ products originated from CO_2_ reduction. The oxidation reaction, critical for artificial photosynthesis, revealed the production of oxygen, H_2_, CO, CH_4_, and C_2_H_6_. H_2_O_2_ formation from water oxidation was confirmed, and under high-intensity ultraviolet light, O_2_ consumption occurred, likely through the formation of other oxygen species. Overall, the results showcase an artificial photosynthesis process using AuIr@InGaN NWs/Si for photocatalytic CO_2_ reduction and water oxidation, introducing an effective APID for direct C^2+^ hydrocarbon synthesis with light, CO_2_, and water as sole inputs.

### Photocatalytic CO_2_ Reduction

Syngas is often produced through the reformation of fossil fuels, which greatly raises atmospheric CO_2_ levels. On the other hand, solar-powered CO_2_ reduction with H_2_O appears to be the best option for dealing with both anthropogenic CO_2_ collection and renewable CO/H_2_ synthesis [168]. However, insufficient photon harvesting and strong electron–hole recombination make direct syngas synthesis from CO_2_ and H_2_O via photocatalysis a significant issue even today [169]. It is very difficult to achieve CO_2_ RR together with H_2_O splitting for tunable CO/H_2_ ratios without bias or sacrificial agents. Reports of photocatalysts show low solar-to-syngas (STS) energy efficiency, non-adjustable H_2_/CO ratios, and poor syngas activity despite advances. Moreover, photoexcited holes are usually not consumed by inexpensive sacrificial agents such as triethanolamine, and oxygen is not liberated during water splitting. While Au is known to catalyze CO_2_ RR to CO, there are still few effective instances of gold-only photocatalysis of CO_2_ RR for syngas synthesis, particularly in the presence of CO_2_, H_2_O, and light. It is critically necessary to address key difficulties in photocatalytic CO_2_ reduction into syngas for practical applications.

**Fig. 11:**
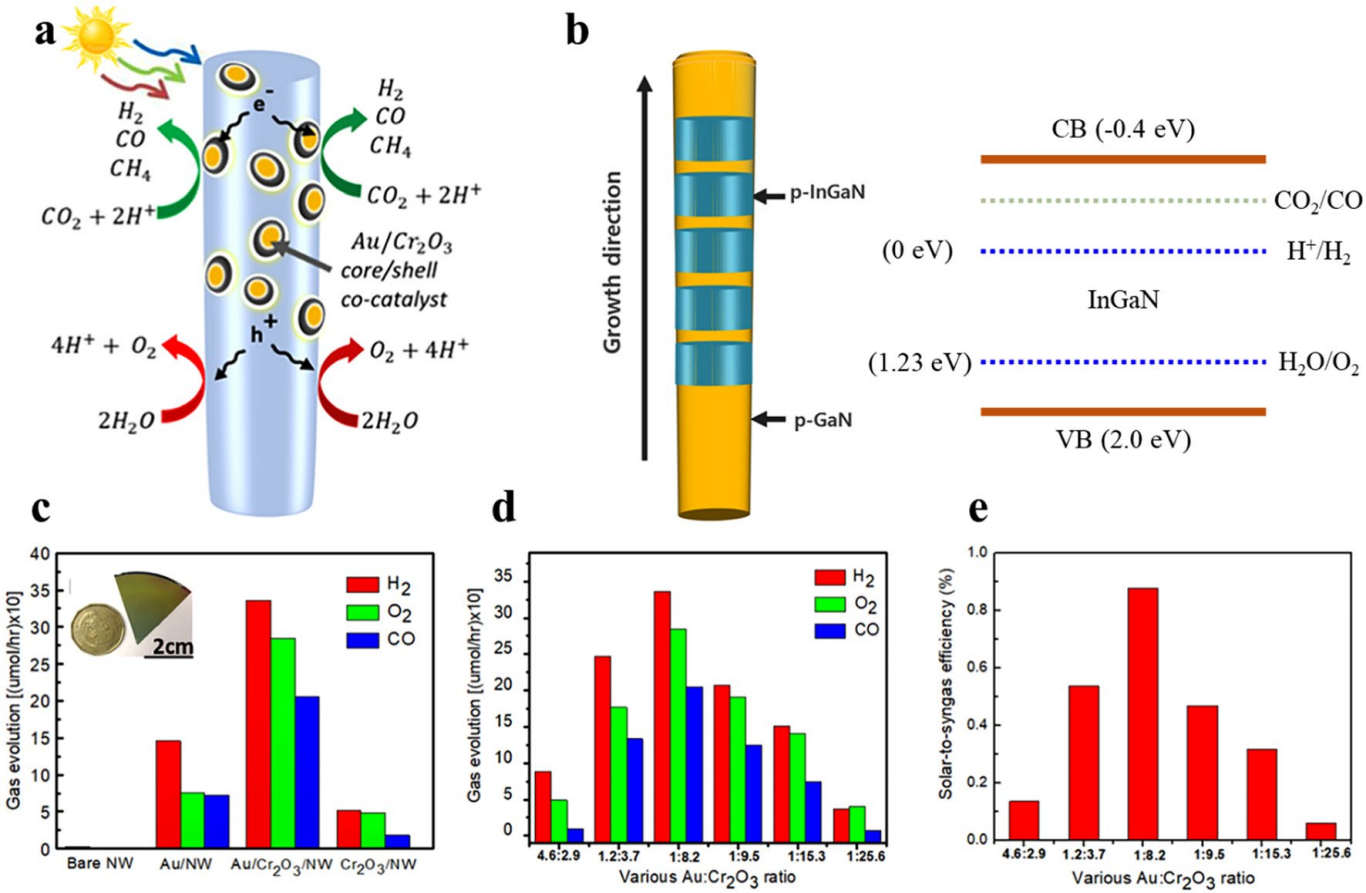
Overview: Au@Cr_2_O_3_-decorated InGaN/GaN NW for CO_2_RR **a** diagram illustrating the complete CO_2_ reduction reaction (CO_2_RR) over Au@Cr_2_O_3_-decorated InGaN/GaN nanowires (NW). **b** Bandgap diagram of InGaN, strategically positioned to cover the redox potentials conducive to overall CO_2_RR toward CO in a water environment. **c** Gas evolution results on nanowires with various cocatalysts, with an inset displaying a typical nanowire sample used in the experiments. **d** Gas evolution results with various Au@Cr_2_O_3_-decorated InGaN/GaN nanowires. **e** Relationship between the surface-to-surface ratio (STS) and Au/Cr_2_O_3_ ratios. Reproduced with permission from Ref. [167]. Copyright 2022 Springer PNAS

In a sealed gas-phase reactor, InGaN/GaN nanowires adorned with Au@Cr_2_O_3_ were utilized for photochemical CO_2_ reduction in distilled water. Electrons and holes were produced when light was applied to the multilayered InGaN/GaN structure. In Fig. 11a, the schematic illustration is displayed. The photogenerated holes were crucial in splitting water to liberate protons, while the electrons participated in the hydrogenation of CO_2_ to produce CO and H_2_. The valence band (VB) and conduction band (CB) energy locations of the photocatalyst matched the redox potentials required for CO_2_ reduction with water oxidation, which is noteworthy (Fig. 11b). It was shown that the lack of catalytic sites in bare InGaN/GaN nanowires resulted in low activity for CO_2_ reduction and the hydrogen evolution reaction (HER) under focused solar irradiation of 1.6 W cm^−2^. But once the Au@Cr_2_O_3_ cocatalyst was added, the semiconductor platform showed a noticeably higher syngas generation rate. The reaction chamber was air-cooled to between 45 and 50 °C in order to reduce the effect of temperature and ensure that thermal effects were minimal. With a preferred H_2_/CO ratio of 1.6:1 and a notable syngas production rate of 1.08 mol g_cat_^−1^ h^−1^ of catalyst per hour under these optimized conditions (Fig. 11c), the system is suited for the production of hydrocarbon fuels and methanol. At a rate of 0.57 mol g_cat_^−1^ h^−1^ of catalyst per hour, virtually stoichiometric O_2_ was produced simultaneously, and very few additional gaseous carbonaceous products—a trace amount of CH_4_ being the only one-were found.

Furthermore, Au/Cr_2_O_3_ outperformed Au and Cr_2_O_3_ alone, suggesting that these substances cooperate to lower CO_2_. By adjusting the Au/Cr_2_O_3_ ratio, the CO + H_2_ activity and the H_2_/CO ratio could be regulated over a broad range (Fig. 11d). At an initial Au/Cr_2_O_3_ ratio of 1:25.6, H_2_ and CO were generated at rates of 0.074 and 0.013 mol g_cat_^−1^ h^−1^ of catalyst per hour, respectively. A high H_2_/CO ratio of 5.5 resulted from this. The CO/H_2_ activity improved with an increase in the Au/Cr_2_O_3_ ratio and peaked at a ratio of 1:8.2 (Fig. 11e), with an STS efficiency of 0.89%. Higher Au/Cr_2_O_3_ ratios, however, were associated with a deterioration in performance; ultimately, the syngas activity fell to 0.20 mol g_cat_^−1^ h^−1^ at a ratio of 4.6:2.9, with a maximum H_2_/CO ratio of 9.2. These findings point to the photocatalyst’s resilience and longevity. Furthermore, the photocatalytic CO_2_ reduction activity was verified at visible light wavelengths (above 400 nm) by observing the evolution of H_2_ and CO from the Au@Cr_2_O_3_-decorated InGaN/GaN nanowires in multiple cycles, with no discernible deterioration. H_2_ and CO evolution rates were measured under visible light at 0.368 and 0.108 mol g_cat_^−1^ h^−1^, respectively. An estimated STS efficiency of roughly 0.39% was obtained, slightly less than the 0.89% obtained under AM 1.5G solar light illumination. This difference was explained by the InGaN/GaN nanowires’ narrow wavelength range (400–507 nm) in visible light, which eliminated some wavelengths. This work offers a carbon–neutral method of producing green syngas, which has the potential to completely transform the traditional methods based on fossil fuels.

### PV-GDE CO_2_ Reduction

**Fig. 12:**
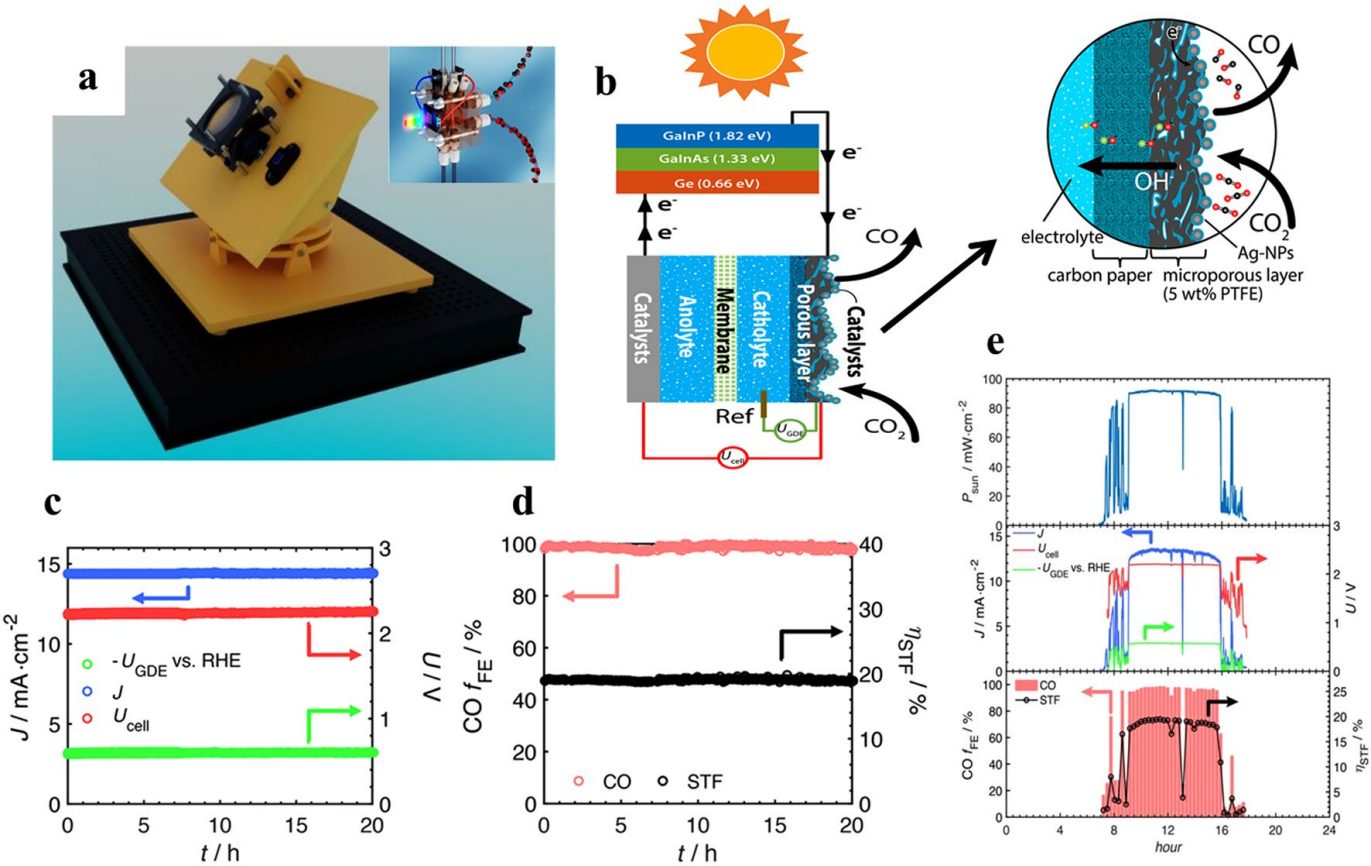
Light-driven photovoltaic-gas diffusion electrode (PV-GDE) measurement. **a** Image of the photoreactor for overall CO_2_ reduction and the solar tracker, with an effective area of 0.31 cm^2^. **b** An illustration of the reverse-assembled GDE cathode cross section and a schematic representation of the wire connection between the triple-junction cell and the gas diffusion electrode (GDE) cell highlight the wetted catalyst and the CO_2_ reduction process. **c** Measurements of cell voltage, current, and GDE potential in relation to the reversible hydrogen electrode (RHE) throughout a 20-h period. **d** Corresponding CO Faradaic efficiency and solar-to-fuel efficiency over the same 20-h duration. **e** Outdoor evaluations of solar-driven PV-GDE in Pasadena, CA, with an effective area of 0.31 cm^2^. Operating current density, cell voltage Ucell, GDE potential UGDE vs RHE, CO Faradaic efficiency, CO, and solar-to-fuel efficiency STF were recorded for a 24-h day cycle. These outcomes were from evaluations of the solar-powered PV-GDE system conducted outside. Reproduced with permission from Ref. [171]. Copyright 2022 Springer, ACS Energy letters

The study included a solar-powered carbon dioxide reduction technique that used a gas diffusion electrode (GDE) that was directly driven by a PV cell [168–170]. An Ag-NP catalyst layer on a reverse-assembled gas diffusion electrode was powered by the energy from a triple-junction solar cell including GaInP, GaInAs, and Ge. The effort led to the development of the first-ever flow-type electrolyte PV-GDE reactor, which has outstanding selectivity and long-term stability. More specifically, a triple-junction photovoltaic (PV) system was directly combined with a gas diffusion electrode (GDE). The GDE was immediately powered by the PV cell’s generated energy to reduce carbon dioxide. Figure 12a shows a picture of the photoreactor, which contains the solar tracker and the CO_2_ reduction system. A photovoltaic (PV) component that tracked the sun was made up of a light-dependent resistor sensor array. On the microporous side of the gas diffusion electrode (GDE) substrate, 0.12 mg cm^−2^ of diluted silver nanoparticles (Ag-NPs) with diameters less than 50 nm were loaded. The GDE was compressed to improve CO_2_-catalyst interaction and gas utilization after gas was supplied to it via an interdigitated electrode flow field. Utilizing a Fumasep FAA-3-50 membrane, the electrolyte (1 M KOH) was separated. The interdigitated electrode on the Ag-NP/carbon paper substrate allowed the current to be applied to the gas diffusion electrode (GDE).

Both the cell potential (Ucell) and the cathode-to-reference electrode potential (UGDE) were measured during operation. The Ag-NP catalyst faced the CO_2_ gas channel in a reverse-assembled GDE, and the system functioned as a “flow-by” GDE with the gas inlet and outflow on the same side. Polytetrafluoroethylene (P_TFE_) was applied to the GDE’s microporous layer in order to stop flooding. In order to separate the liquid and gas phases and control the pressure differential between the CO_2_ stream and the aqueous electrolyte, needle valves were added into the gas and liquid output streams. In the laboratory, the system’s stability and efficiency were assessed with AM 1.5G solar illumination simulation. The system demonstrated an average CO production rate of 2.3 mg h^−1^ and an average Faradaic efficiency for CO of 99 ± 2% over a 20-h (Fig. 12c). We did not see any decline in performance. During the 20-h operation, the average solar-to-CO efficiency was calculated to be 19.1 ± 0.2%, while the average energy efficiency, or η_GDE_, was found to be 59.4 ± 0.6% (Fig. 12d).

Full-day outdoor testing with online gas product analysis was done to determine the solar-to-fuel efficiency over the course of the day, as indicated in Fig. 12e. The system operated at 2.20 V for cell voltage and − 0.57 V for GDE potential under full sun light. A solar-to-fuel conversion efficiency of 18.7 ± 1.7% and a Faradaic efficiency of 96 ± 8% were measured during the best six hours of the day. The average daily solar-to-fuel conversion efficiency was 5.8%. The estimated daily rate of CO generation under real outdoor sunshine circumstances was 15 mg day^−1^. The device could produce 0.5 kg of CO each day if it were scaled up to 1 m^2^. By breaking the previous record for directly solar-driven carbon dioxide reduction efficiency, the PV-GDE system demonstrated its exceptional efficiency and dependability as a solar CO_2_ conversion tool. In conclusion, the system’s noon solar-to-CO conversion efficiency under outside sunshine circumstances was 18.7%.

## Outlook and Future work

Addressing the growing energy issue and advancing sustainability require the use of H_2_ and CO_2_ as environmentally benign substitutes for fossil fuels. This study scrutinizes numerous III–V semiconductor materials with the aim of enhancing effective H_2_ and CO_2_ production/reduction in renewable energy systems. This overview of advancements in CO_2_ reduction processes, water electrolysis, photoelectrochemical and photocatalytic water splitting, and other related fields is to achieve high performance, low cell voltage, and long-term stability through creative photocatalytic electrocatalyst design (Table 5). Ensuring broad light absorption in systems using photoelectrodes is crucial for achieving high yields of target products. Despite notable advancements in green energy systems for CO_2_ reduction and water splitting, challenges still exist. While III–V semiconductor materials and cocatalysts exhibit effectiveness in water electrolytes, their performance is impeded by challenges such as photogenerated electron–hole recombination, limiting the efficient utilization of charge carriers. Therefore, it is crucial to carefully regulate the features of charge carrier transport, surface band bending, and electrical conductivity when employing III–V semiconductor materials. Despite the large number of cocatalysts that have been developed, they frequently cannot satisfy the high current density and long-term stability requirements of industry. For industrial applications, it is therefore essential to create electrodes for HER and OER that are big in size, have a lot of active sites, and exhibit excellent stability. Various renewable energy sources, including solar, thermal, wind, and water, can be harnessed to generate electrical energy through diverse methods. For instance, the Rh/Cr_2_O_3_/Co_3_O_4_–InGaN/GaN NWs PEC water splitting explores the viability of temperature-dependent photocatalytic oxygen evolution reactions (OWS) in tap water and seawater. Coupling a high-efficiency triple-junction solar cell with two electrolyzers in series in PV-EC water splitting proves effective, minimizing excess voltage generated by multi-junction solar cells and optimizing high-efficiency PV utilization for water splitting. In PV-PEC water splitting, precise control and management of water flow rates are crucial for achieving thermal integration synergistically. Demonstrated effective, the control strategies dampen variations in solar radiation-induced hydrogen and heat production dynamics. PEC/PEC water splitting, utilizing a dual-photoelectrode device with a parallel illumination scheme and the flexible bandgap engineering of metal-nitride nanowire structures, enhances power conversion efficiency, functioning in both light and dark conditions. The Pt/GaN/Si assembly serves as a catalytic electrode, promoting reactions and demonstrating energy-saving capabilities with the usage of seawater. Catalyst-free InGaN nanowires in water splitting exhibit a unique pyramidal nanostructure with varied indium compositions and a specially designed quantum sliding energy band structure. The broad light absorption range, from UV to NIR, enhances power conversion efficiency. PEC/PV cell efficiency modeling guides the selection of bandgaps in tandem absorber water splitting devices. The innovative multi-junction (IMM) device architecture validates isoefficiency modeling, enhancing power conversion efficiency. Flue gas CO_2_ reduction, employing CuS NPs on GaN nanowires, enables the conversion of real CO_2_ gas into chemicals, offering a promising route for cost-effective, high-efficiency solar fuel production. In the case of high-purity CO_2_ gas reduction, the mediation of gold by iridium breaks the bottleneck of C–C coupling during light-mediated CO_2_ reduction, illustrating a method for C_2_ alkane synthesis from CO_2_ and H_2_O without external bias or sacrificial agents. Photocatalytic CO_2_ reduction with InGaN/GaN nanowires achieves a record-high solar-to-syngas efficiency, providing tunable H_2_/CO ratios through controlled engineering of Au@Cr_2_O_3_. Isotopic testing confirms that syngas originates from CO_2_ reduction, simultaneously evolving stoichiometric oxygen from water splitting. The reverse-assembled PV-GDE sets a new efficiency record for direct solar-driven CO_2_ reduction, exemplifying a highly efficient and stable device for solar CO_2_ conversion. Even though a variety of water splitting devices have made great strides, more work needs to be done in this area before large-scale, useful clean energy systems can be applied. The review is to stimulate additional efforts toward developing innovative green energy systems for the production of hydrogen, guaranteeing an economical, environmentally friendly, and long-lasting conversion process in real-world applications.

**Table 5:** Summarized performance for PEC, PV-EC, and PEC-PV systems

Approaches for solar H_2_ generation and CO_2_ reduction	Advantages	Challenges
PEC water splitting and CO_2_ reduction	- Highly efficient H_2_ generation - Cocatalysis increased device efficiency - Highly efficient CO_2_ reduction - Low environmental impact	- High cost - High complexity - Without Cocatalysis - Light absorption (water and light scattering at bubble interface) - Unsatisfactory stability
PV-EC water splitting and CO_2_ reduction	- Mature technology and commercially viable - Splitting elements do not affect light absorption - Easily scalable - Low resistance through electrolyte - Highly efficient - Long lifetime	- High cost - High complexity - Losses through transparent conductor and electrical wires - Relatively high environ-mental impact for manufacturing the PV cell - The need for separator
PV water splitting	- Highly efficient - Relatively low cost - Low environmental impact - Eliable and viable	- Requirement for an effective solar collector - Light absorption (water and light scattering at bubble interface) - Unsatisfactory stability
